# Pre-conception and prenatal alcohol exposure from mothers and fathers drinking and head circumference: results from the Norwegian Mother-Child Study (MoBa)

**DOI:** 10.1038/srep39535

**Published:** 2016-12-23

**Authors:** Luisa Zuccolo, Lisa A. DeRoo, Andrew K. Wills, George Davey Smith, Pål Suren, Christine Roth, Camilla Stoltenberg, Per Magnus

**Affiliations:** 1 MRC Integrative Epidemiology Unit, School of Social and Community Medicine, University of Bristol, Bristol, UK; 2 Department of Global Public Health and Primary Care, University of Bergen, Bergen, Norway; 3 School of Clinical Sciences & School of Oral and Dental Sciences, University of Bristol, Bristol, UK; 4 Division of Epidemiology, Norwegian Institute of Public Health, Oslo, Norway; 5 Department of Epidemiology, Columbia University, New York, USA; 6 Nic Waals Institute, Lovisenberg Hospital, Oslo, Norway; 7 Director’s Office, Norwegian Institute of Public Health, Oslo, Norway

## Abstract

Although microcephaly is a feature of Fetal Alcohol Syndrome, it is currently unknown whether low-to-moderate prenatal alcohol exposure affects head circumference. Small magnitude associations reported in observational studies are likely to be misleading due to confounding and misclassification biases. Alternative analytical approaches such as the use of family negative controls (e.g. comparing the effects of maternal and paternal exposure) could help disentangle causal effects. We investigated the association of maternal and paternal alcohol drinking before and early in pregnancy with infant head circumference, using data from 68,244 mother-father-offspring trios from the Norwegian Mother and Child Cohort Study (MoBa) (1999–2009). In analyses adjusted for potential confounders, we found no consistent pattern of association between maternal or paternal alcohol intake before or during pregnancy and offspring head circumference modelled as a continuous outcome. However, we found higher odds of microcephaly at birth for higher paternal, but not maternal, alcohol consumption before pregnancy, and similar but weaker effect estimates for first trimester drinking. Associations with paternal drinking before pregnancy were unexpected and should be regarded as hypothesis generating, until independently replicated, although potentially important given the absence of guidelines on safe drinking levels for men in couples trying for a pregnancy.

Alcohol consumed during pregnancy crosses the placenta and heavy use has long been known to cause Fetal Alcohol Syndrome (FAS)^1^. Both FAS and Fetal Alcohol Spectrum Disorder are characterised, amongst other features, by microcephaly and intellectual disability^2^. The neurodevelopmental effects of prenatal exposure to low-to-moderate levels of alcohol remain unclear, with some epidemiological findings suggesting no or even modest protective associations^3^,^4^,^5^,^6^,^7^,^8^,^9^. Given the potential for residual confounding and misclassification biases, associations of such small magnitude reported in observational studies are likely to be misleading^10^, as indicated by recent natural experiment type studies on cognition^11^, academic achievement^12^, and other long-term outcomes^13^.

Head circumference at birth is a marker for brain development throughout gestation^14^ and predictive of cognition in later life^15^, especially for more vulnerable children (born low birth weight, premature, or with intrauterine growth restriction)^16^ and those exposed to alcohol during pregnancy^17^. It is currently unknown whether in general population terms, head size is associated with varying levels of prenatal alcohol exposure, given the scant epidemiological evidence^18^,^19^,^20^,^21^, and again the potential for residual confounding.

Family-based designs could produce better estimates of causal associations, disentangling the confounding effects of shared genes and environment from the true causal effect of prenatal alcohol exposure. A powerful approach when prenatal exposure data are available for both parents is to compare the association of maternal exposure with offspring outcomes to that of paternal exposure with the same outcomes^22^. Under the assumption that maternal and paternal associations are similarly, if not equally, confounded by shared genes and shared environment, the comparison can be informative as outlined by previous ‘proof-of-principle’ work on maternal smoking in pregnancy and offspring birth weight^22^.

We therefore conducted maternal-offspring and paternal-offspring analyses using data from MoBa, a very large pregnancy cohort from Norway. Specific aims were: 1) to investigate the association of maternal drinking in the months before and early in pregnancy (most sensitive periods to alcohol exposure^20^,^23^) with infant head circumference; and 2) to compare effect estimates to those of paternal drinking in the same periods on infant head circumference. This was done in an attempt to disentangle real biological effects (e.g. intrauterine for the maternal exposure relative to early pregnancy and paternal-line effects via gametes modification for the paternal exposure before conception), from shared environmental and genetic effects, expected to be of similar magnitude for both parental exposures.

## Results

After excluding twins (n = 3,813), we identified 104,983 mother-father-child trios. Further exclusions were based on: gestational age <32weeks (n = 1,099), neonatal deaths (n = 322), implausible head circumference measurements (n = 87), missing head circumference at birth (n = 2,112), missing data on maternal (n = 8,596) or paternal alcohol use (n = 24,523). Of the remaining 68,244 trios, 46,178 trios had data on the full set of confounders.

### Alcohol drinking patterns: mother-father and before-during pregnancy correlations

Maternal and paternal drinking were more strongly correlated before than during pregnancy, with the highest correlation values for non-drinkers (r = 0.448 before pregnancy, and r = 0.111 during) and heavy drinkers (5+ units, r = 0.352 before and 0.106 during) (Supplementary Tables S1 Vs S2). On the other hand, drinking in moderation (1–2 units) correlated positively before but not during pregnancy (r = 0.235 and r = 0.019).

**Figure 1:**
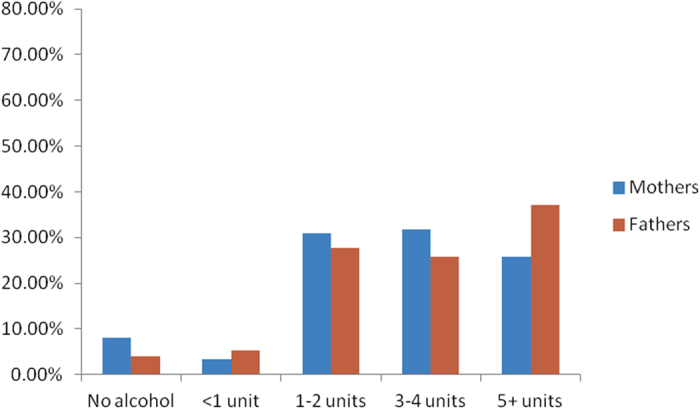
Distribution of Alcohol Consumed per Occasion by Mothers and Fathers in the Months Before Pregnancy (68,244 Eligible Trios, MoBa data, Norway, 1999–2009).

**Figure 2:**
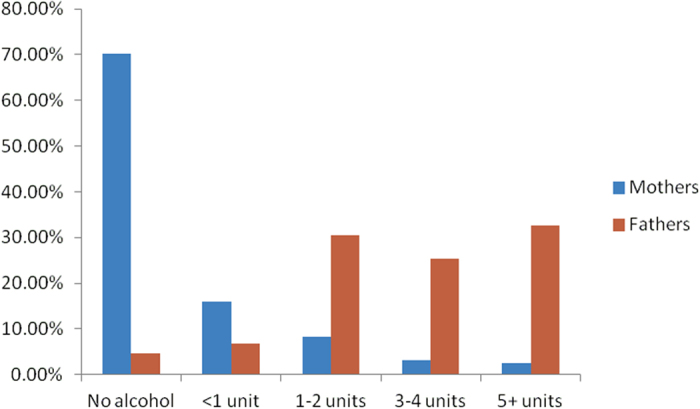
Distribution of Alcohol Consumed per Occasion by Mothers and Fathers in the First Trimester of Pregnancy (68,244 Eligible Trios, MoBa data, Norway, 1999–2009).

Fathers consumed more drinks per occasion than mothers, particularly in the first trimester of pregnancy as their partner’s pregnancy altered their drinking behaviour only marginally (Figs 1 and 2, Supplementary Tables S1–S2). Fathers tended to report stable drinking behaviours, with very strong positive correlations along the diagonal, for the periods before and during their partner’s pregnancy, and moderately strong negative correlations between reducing from 5+ units or increasing to 5+ units (Supplementary Table S3).

As expected, many mothers markedly reduced the amount of alcohol consumed at any occasion (Supplementary Table S4), presumably from pregnancy recognition. 8% of mothers reported abstaining from alcohol in the first trimester (Fig. 2), compared to only 70% who abstained during the three months preceding pregnancy (Fig. 1). However, consuming 5+ units in the first trimester showed a correlation of 0.241 with consuming the same amount before pregnancy (Supplementary Table S4), the highest between-category correlation, indicating that some heavy drinking habits remained even during the pregnancy. Maternal drinking behaviour generally tracked later on in the pregnancy, into the second and third trimester (Supplementary Tables S5–S6).

### Distribution of confounders by exposure and outcome

We checked the key assumption of this study design, that confounders are similarly distributed across categories of maternal and paternal alcohol consumption, and reported the results in Supplementary Information available online (Supplementary Tables S7–S10, Figures S1–S2). There was evidence of association with offspring head circumference at birth (and at 3 months) for most of the potential confounders identified (Supplementary Tables S11 Vs S12).

### Maternal and paternal alcohol–outcomes associations

We found no consistent, robust patterns of association between maternal or paternal alcohol intake before or during pregnancy and offspring head circumference, at birth or 3 months post-partum, modelled as a continuous outcome (Tables 1 and 2). The associations were precisely estimated but close to the null, with only very modest evidence of a small detrimental association of increasing paternal alcohol use with head circumference at 3 months, and didn’t change after adjusting for the other parent’s drinking and a comprehensive set of confounders (Tables 1 and 2). However, we found evidence of higher odds of being born with microcephaly for higher paternal, but not maternal, alcohol consumption, following a trend when comparing 1–2 units Vs none (OR 1.48, 95% CI 0.77–2.84), to 3–4 units Vs none (OR 1.64, 95% CI 0.85–3.16), to 5+ units Vs none (OR 1.93, 95% CI 1.01–3.70) (full model, Table 3). The effect estimates were similar across the three models with varying degrees of confounder adjustments, and seemed stronger for paternal drinking before pregnancy compared to drinking in the first trimester of pregnancy (e.g. OR (5+ units Vs none) 1.93 (1.01–3.70) and 1.36 (0.81–2.28) for before and during pregnancy, respectively, and *P*_trend_(before) 0.036, *P*_trend_(during) 0.056). There was also some evidence that mothers consuming <1drink/occasion during pregnancy were less likely to have offspring with microcephaly at birth compared to non-drinkers (OR 0.68, 95% CI 0.50–0.94), but no trend was noted (*P*_trend_ 0.545) (Table 3). For the analyses of microcephaly at 3 months post-partum, point estimates were in the same direction to those found for the outcome at birth (Table 4).

### Sensitivity analyses

Sensitivity analyses replacing dose per occasion exposures with cumulative (average units/week) exposures revealed very similar patterns of associations compared to the main analyses (Supplementary Tables S13–S16). In particular, paternal pre-pregnancy alcohol consumption was associated with even higher odds of microcephaly at birth compared to those found for the dose per occasion analyses, with higher odds and a sharper dose-response effect for pre-pregnancy compared to first-trimester consumption (Supplementary Table S15, full model). These estimates were attenuated in relation to the outcome measured at 3 months (Supplementary Table S6). One new finding of these analyses compared to the main analyses was some evidence of a protective effect for any level of maternal alcohol consumption before pregnancy on the odds of microcephaly (ORs in the range of 0.61 to 0.73, Supplementary Table S15, full model). However, this did not follow a dose-response pattern and the apparent associations disappeared when examining the outcome at 3 months and relative to maternal consumption during the first trimester (Supplementary Tables S15–S16).

Sensitivity analyses were also conducted to check the robustness of the association of paternal alcohol pre-pregnancy with odds of microcephaly at birth and 3 months.

Results were robust to the exclusion of some father-child pairs based on pre-specified criteria (see methods for a full description, and Supplementary Table S17, for results). Restricting the analyses to observations with complete data for all confounders attenuated effect estimates, more so for the outcome measured at birth compared to the outcome measured at 3 months, however 95% CIs overlapped substantially between the main analysis and sensitivity analysis (complete case sample Vs eligible sample) (Supplementary Table S18).

Additional adjustments for fetal presentation at birth didn’t change the results (Supplementary Table S19). Changing the threshold for microcephaly to the bottom 5% of the z-score distribution, and then the bottom 10% resulted in effect estimates attenuated towards the null more and more, in particular for paternal alcohol intake before pregnancy (results available from the authors upon request).

## Discussion

Our a priori hypothesis was that in the presence of a truly biological intrauterine effect, maternal alcohol use during pregnancy would be associated with neonatal head circumference, whereas paternal alcohol use wouldn’t, or the association would be of smaller magnitude at least. Vice versa, associations of similar magnitude for both parental exposures would indicate the presence of confounding, and observing differences in head circumference by paternal, but not maternal, alcohol use would be consistent with pre-conception effects through the paternal germ-line. In this study, we didn’t find robust evidence for a maternal effect, not even for higher doses of prenatal alcohol exposure, and instead uncovered suggestive evidence of a possible association of paternal alcohol use before conception on offspring microcephaly. This doesn’t necessarily rule out the existence of a (probably modest) intrauterine effect for certain levels of maternal drinking, which the study could have been underpowered to reveal due to the vast majority of mothers quitting alcohol upon pregnancy recognition.

Only one of many systematic reviews on the effects of maternal-line prenatal alcohol exposure included head circumference as an outcome, and found limited evidence of association^24^. Four more recent cohort studies also reported on this. A large Dutch study showed no evidence of association with head circumference from ultrasound scans^19^, and another large US-based birth cohort also found little evidence of detrimental effects but a suggestive reduction in the odds of microcephaly associated with maternal drinking in the first trimester (generally after pregnancy recognition)^21^. However, two smaller studies from the US and Spain reported some evidence of a dose-response relationship between maternal drinking in the first trimester and increased risk or microcephaly^20^ and reduced head circumference^18^. Our results are in concordance with the former two studies reporting predominantly null findings, and the distributions of maternal drinking in pregnancy are similar too, whereas the two smaller studies included a larger proportion of moderate-to-heavy drinkers, which might have driven the results.

We were unable to find epidemiological evidence relating specifically to paternal (pre-conception) alcohol use and offspring head circumference. However, previous studies in humans have shown evidence of paternal-line effects in relation to fetal growth^25^, mild cognitive impairment^25^, and even spontaneous abortions^26^. Animal models have shown that offspring sired by ethanol-exposed males exhibited stunted growth compared to controls^27^, with studies of acute alcohol exposure showing more consistent results than studies of chronic exposure^28^. Moreover, evidence of alcohol effects on sperm DNA^29^ including deterioration of sperm parameters^30^, alongside evidence of demethylation of normally hypermethylated imprinted regions in sperm DNA in heavy drinkers^31^,^32^, raise the possibility of a paternal involvement in FASD through either or both de-novo mutations in sperm DNA or epigenetic mechanisms.

The present study has several strengths. It is a population-based study with prospectively collected information on alcohol behaviour in pregnancy for both parents, and on many potential confounders, ruling out recall bias and reducing the risk of major confounding. The sample size is very large, providing good statistical power especially for the paternal analyses, however the large proportion of women quitting alcohol in pregnancy could have resulted in limited statistical power to uncover subtle maternal effects. Data on alcohol use at different times before and during pregnancy for both parents helps with the interpretation of parent-of-origin effects (biological plausibility of paternal effects if associations with alcohol before pregnancy are larger than those with alcohol during pregnancy). Consistent results across analyses using dose per occasion and cumulative weekly dose as exposures lend confidence in our results. Outcome data are available on the whole eligible cohort, through linkage with a national registry, allowing us to check for the extent of selection bias. Outcome data are available both at birth and 3 months post-partum, which allowed us to validate the main analyses against confounding by fetal presentation and/or mode of delivery causing misshaped heads. None of the analyses were adjusted or stratified by gestational age, to avoid introducing selection (collider) bias^33^.

Regarding confounding, our analytical approach of comparing maternal-paternal associations is particularly useful in situations where the outcome has a large genetic and/or shared environmental component, and a comparatively smaller non-shared environmental component. In our case, this study design is particularly appropriate since head circumference at birth and in early life has been shown to be markedly heritable^34^, with genetic contributions from both parents playing a role^35^, and influenced by the shared environment^35^. Moreover, empirical checks shown here confirm that in this population maternal and paternal alcohol consumption display similar associations with a number of important confounders. This implies that it is unlikely that the maternal and paternal analyses are affected by radically different extents of residual confounding, and further validates this as a negative control method. However, it is always possible even if not very likely, that some degree of residual confounding exists in any observed association – in this case that between the outcome and paternal drinking.

Self-selection into the cohort and (selective) non-response to questionnaire items could lead to biased results in analyses based on the sub-sample with complete data on all covariates. However, no evidence of selection bias was found in a study comparing exposure-outcome associations based on the entire population of Norway Vs the MoBa cohort^36^. Comparisons included the smoking-low birth weight association, which is similar in nature to our drinking-small head circumference analysis. In sensitivity analyses comparing associations between an analysis of the eligible sample vs. study sample (with complete data), point estimates were closer to the null in the former, however confidence intervals were largely overlapping.

Recall bias is in principle also possible, since the mothers filled in the information about their alcohol use just after the 17 weeks routine ultrasound scan (their first scan in the Norwegian healthcare system). However, since the purpose of this scan is to date the pregnancy and identify major abnormalities, and not to benchmark fetal growth against external references, only very few fetuses would have been identified as suffering from major anomalies. Those pregnancies would have also been at high risk of termination and therefore they would have been excluded from this study, therefore minimising the impact that recall bias might have on our findings even further. As for fathers, we believe their reports are even less likely to suffer from recall bias, since they are much less likely to attribute fetal abnormalities to their own drinking.

Another limitation in interpreting the difference between maternal and paternal association of pre-pregnancy drinking with microcephaly lies in the slightly different methods of exposure assessment, with fathers being asked about their drinking over the 6 months preceding the pregnancy, and mothers over 3 months only. Whereas it is possible that mothers reduce their alcohol consumption in the weeks and months leading to a pregnancy in an effort to improve fertility and minimise fetal harm, fathers are much less likely to do so and therefore their drinking before the pregnancy is likely to be similar whether it refers to 6 or 3 months before the pregnancy and the results for fathers are therefore likely to be similar to those that would be obtained by having asked them exactly the same question as the mothers.

International guidelines on “safe limits” of drinking in pregnancy vary widely ( http://www.icap.org/Table/InternationalGuidelinesOnDrinkingAndPregnancy). This area is of growing public health importance^37^, given the significant risk of fetal exposure especially in early gestation^20^ since many pregnancies are unplanned (up to 40% in the UK alone^38^) and alcohol use (including binge drinking) is prevalent and increasing among women of reproductive age^39^,^40^. Even more importantly, currently there are no guidelines on safe drinking levels for men in couples trying for a pregnancy, or indeed sperm donors, but if the emerging evidence on paternal effects was to be confirmed, new guidelines will need to be issued to the public.

In conclusion, we found evidence of higher odds of being born with microcephaly for higher paternal, but not maternal, alcohol consumption, in particular relative to the period before conception. Although consistent with several lines of evidence from animal models, our suggestive results of an association between paternal drinking and head size, in particular for vulnerable neonates, is to be considered hypothesis generating, until replicated in independent epidemiological studies preferably using other approaches aimed at strengthening causal inference in birth cohorts^41^.

## Methods

### Participants

MoBa is a prospective population-based pregnancy cohort study conducted by the Norwegian Institute of Public Health^42^. Participants were recruited from all over Norway from 1999–2008, and 38.5% of invited women consented to participate. The cohort includes just over 108,000 children, 90,700 mothers and 71,500 fathers. Follow-up is conducted by questionnaires at regular intervals and by linkage to national health registries. The current study is based on version 7 of the quality-assured data files released for research in June 2012. Informed consent was obtained from each MoBa participant upon recruitment. Further details are available on the study website – www.fhi.no/moba-en. We restricted the study to singleton live born children whose both mother and father had provided information on alcohol consumption in the prenatal period.

### Measurement of alcohol intake

Alcohol consumption before and during pregnancy was assessed through questionnaires completed by mothers around 17 and 30 gestational weeks and 6 months post-partum and by fathers around 17 gestational weeks. Mothers were asked about their drinking in the last 3 months before becoming pregnant (questionnaires at 17 and 30 weeks), and in the three pregnancy trimesters (first: questionnaires at 17 and 30 weeks, second and third: questionnaires at 30 weeks and 6 months post-partum), and fathers about the last 6 months before their partner’s pregnancy, and during the pregnancy up to approximately week 18. Questions on drinking frequency and average number of alcohol units per sitting were asked at all of the time points. Units were defined as corresponding to 1.5 cl. pure alcohol (U.S. units), equivalent to 1 bottle/can energy drink or cider, 1 glass (1/3 litre) beer, 1 wine glass red or white wine, 1 sherry glass or other fortified wine, 1 snaps glass spirits or liqueur.

Alcohol information across the questionnaires was standardised for internal consistency. Where the mother reported alcohol intake for the same time period in two consecutive questionnaires, we used the highest reported value.

The main exposure variable was the average alcohol dose per occasion, measured in units/sitting before and during pregnancy (up until week 17) for both mothers and fathers, and then categorised as “non-drinker”, “<1 unit”, “1–2 units”, “3–4 units”, “5+ units”. Cumulative exposure defined as average drinks per week was also used in sensitivity analyses. This was derived from average dose times frequency of consumption, and categorised as “non-drinker”, “<1 unit/week”, “1–2 units/week”, “3–4 units/week”, “5+ units/week”. Non-drinkers were chosen as the reference category in all analyses, because comparisons to this group would be easier to interpret. This category included only individuals who reported to never drink alcohol at a particular time-point (and to never binge drink).

### Measurement of infant head circumference

Head circumference, weight and length routinely measured at birth were available through record linkage with the Norwegian Medical Birth Registry^43^. Gestational age was calculated as the interval between delivery date and last menstrual period, if within 14 days from the estimate based on first trimester ultrasound, and otherwise it was based on said ultrasound, available for 98.2% of MoBa participants (dates from birth registry data). The main outcome variable was defined as sex-standardised head circumference (expressed as standard deviation [SD] scores), based on the distribution of all MoBa newborns by sex (both those included in these analyses and those ineligible). Additionally, since some infants’ head is misshaped at birth due to fetal presentation and/or delivery mode, we also considered SD scores of head circumference measured at approximately 3 months post-partum (data originally from child health records, transcribed to the questionnaire completed by mothers 6 months post-partum). Since the age at clinic visit when these measurements were taken varied greatly between 6 weeks and 6 months, we standardised the measurements for age to make them all comparable. Because the relationship between head circumference and age during infancy is nonlinear and the variance increases with age, we calculated sex-specific age-adjusted SD scores of head circumference after regressing head circumference on age at clinic visit and age squared, separately for each sex and each gestational week of birth. Finally, we dichotomised the outcomes to study the more vulnerable extreme of the distributions, and derived a microcephaly definition for newborns with an SD score <−2 (the bottom 2.275% of the distribution), and a similar one for 3-month olds with an SD score <−2^44^.

### Potential confounders

Many factors were considered as possible confounders of the association of prenatal alcohol exposure with head circumference at birth. These included: year of birth, folic acid use around conception^45^, whether the pregnancy was planned, maternal diabetes (pre-conception diabetes or gestational diabetes), parity, ethnicity (gleaned from whether other languages were spoken alongside Norwegian), financial strain, and maternal and paternal age, height, body-mass index (BMI), gross income, education, and smoking/drug use in pregnancy.

### Ethics

Informed consent was obtained from each MoBa participant upon recruitment. All data collection, storage, management and analysis were performed in accordance with relevant guidelines and regulations. The establishment and data collection in MoBa has obtained a licence from the Norwegian Data Inspectorate (01/4325) and approval from The Regional Committee for Medical Research Ethics (S-97045, S-95113).

### Statistical analysis

We investigated the (between-parent) concordance and (within-parent) stability of alcohol consumption around the index pregnancy through correlation matrices.

We studied the distribution of selected confounders by level of maternal and paternal alcohol dose/occasion (before and during pregnancy) univariate regression models, and by comparing cumulative density functions of maternal alcohol by levels of the confounders to those of paternal alcohol by levels of the same confounders. This was done to empirically check the assumption (central to this negative control study) that shared environmental factors relate to both maternal and paternal exposure levels in a similar way.

We examined confounders-outcome associations through another series of univariate linear regressions of head circumference SD scores.

We fitted three models to all exposure-outcome combinations (4 exposures: maternal and paternal alcohol dose/occasion before and during pregnancy - 4 outcomes: sex-standardised head circumference and microcephaly at birth and at 3 months): 1) crude, only including maternal or paternal exposure as outcome predictor; 2) mutually-adjusted, additionally adjusted for the other parent’s exposure (to account for assortative mating), and 3) full, additionally adjusted for the other parent’s exposure and for the following confounders: year of birth, folic acid use around conception, whether the pregnancy was planned, parity, ethnicity, financial strain, maternal and paternal age, height, BMI, gross income, education, smoking in pregnancy, and maternal drug use in pregnancy. We express all results as mean differences in SD scores or odds ratios (ORs) of microcephaly, compared to the category of non-drinkers, and present them with 95% confidence intervals (CI).

We performed tests for linear trend to investigate whether any of the exposure-outcome combinations followed a dose-response pattern.

We performed sensitivity analyses using cumulative exposure measures instead of dose per occasion measures. We also conducted sensitivity analyses to check the robustness of suggestive results. We investigated whether results were robust to the exclusion of the following: a) Congenital malformations; b) Maternal pre-eclampsia, gestational diabetes, or unknown follow-up of child; c) outliers (head circumference more than 4 SD away), or mother-reported ‘abnormal head circumference’; d) C-section deliveries; e) maternal at risk drinking in the year prior to pregnancy (assessed through the T-ACE screening questionnaire^46^). We additionally investigated whether results were explained by restricting the analyses to the observations with complete data on all confounders, by foetal presentation at birth, or by the choice of a different threshold to define microcephaly (e.g. bottom 5% and 10% of the distribution rather than the bottom 2.275%).

All statistical tests were 2-sided. Analyses were conducted using Stata 13.

## Additional Information

**How to cite this article**: Zuccolo, L. *et al*. Pre-conception and prenatal alcohol exposure from mothers and fathers drinking and head circumference: results from the Norwegian Mother-Child Study (MoBa). *Sci. Rep.* **6**, 39535; doi: 10.1038/srep39535 (2016).

**Publisher's note:** Springer Nature remains neutral with regard to jurisdictional claims in published maps and institutional affiliations.

## Supplementary Material

Supplementary Information

## Figures and Tables

**Table 1:** Maternal and Paternal Average Alcohol Dose per Occasion and Head Circumference at Birth – Differences in SD Scores (Beta) and 95% Confidence Intervals (CI), MoBa data, Norway, 1999–2009.

	Mother			Father		
	Beta	95% CI	*P*	Beta	95% CI	*P*
Before pregnancy						
Crude model						
Non drinker	Ref			Ref		
<1 unit	−0.04	−0.09, 0.01	0.124	−0.01	−0.06, 0.04	0.625
1–2 units	0.01	−0.02, 0.04	0.714	0.02	−0.02, 0.06	0.429
3–4 units	−0.01	−0.04, 0.02	0.394	0.01	−0.04, 0.05	0.775
5+ units	−0.01	−0.04, 0.02	0.672	−0.04	−0.08, 0.00	0.073
Mutually adjusted model						
<1 unit	−0.04	−0.09, 0.01	0.131	−0.01	−0.07, 0.04	0.627
1–2 units	0.00	−0.03, 0.04	0.824	0.01	−0.03, 0.06	0.531
3–4 units	−0.00	−0.04, 0.03	0.833	0.00	−0.04, 0.05	0.899
5+ units	0.02	−0.02, 0.05	0.362	−0.04	−0.09, 0.00	0.059
Fully and mutually adjusted model^∗^						
<1 unit	−0.03	−0.08, 0.02	0.230	−0.02	−0.07, 0.04	0.547
1–2 units	0.01	−0.02, 0.05	0.415	−0.00	−0.05, 0.04	0.831
3–4 units	0.01	−0.03, 0.04	0.732	−0.00	−0.05, 0.04	0.915
5+ units	0.03	−0.01, 0.06	0.120	−0.02	−0.07, 0.02	0.293
In the first trimester of gestation						
Crude model						
<1 unit	0.00	−0.02, 0.02	0.978	−0.00	−0.05, 0.04	0.931
1–2 units	−0.03	−0.06, −0.00	0.047	0.03	−0.01, 0.06	0.192
3–4 units	0.02	−0.02, 0.07	0.308	0.02	−0.02, 0.05	0.440
5+ units	−0.03	−0.08, 0.03	0.295	−0.02	−0.06, 0.02	0.380
Mutually adjusted model						
<1 unit	−0.00	−0.02, 0.02	0.841	−0.00	−0.05, 0.04	0.968
1–2 units	−0.03	−0.06, −0.00	0.035	0.03	−0.01, 0.07	0.160
3–4 units	0.03	−0.02, 0.07	0.228	0.02	−0.02, 0.06	0.378
5+ units	−0.02	−0.07, 0.04	0.503	−0.02	−0.05, 0.02	0.432
Fully and mutually adjusted model^#^						
<1 unit	0.00	−0.02, 0.02	0.896	−0.00	−0.05, 0.04	0.860
1–2 units	−0.02	−0.05, 0.01	0.124	0.01	−0.03, 0.05	0.548
3–4 units	0.06	0.02, 0.11	0.009	0.01	−0.03, 0.05	0.666
5+ units	0.01	−0.04, 0.06	0.729	−0.01	−0.04, 0.03	0.728

^∗^Test for trend maternal and paternal alcohol intake in full model: p = 0.067 and p = 0.082.

^#^Test for trend maternal and paternal alcohol intake in full model: p = 0.267 and p = 0.201.

**Table 2:** Maternal and Paternal Average Alcohol Dose per Occasion and Head Circumference at 3 Months Post-Partum – Differences in SD Scores (Beta) and 95% Confidence Intervals (CI), MoBa data, Norway, 1999–2009.

	Mother			Father		
	Beta	95% CI	*P*	Beta	95% CI	*P*
Crude model						
Non drinker	Ref			Ref		
<1 unit	0.01	−0.05, 0.08	0.705	−0.01	−0.08, 0.05	0.730
1–2 units	0.00	−0.04, 0.04	0.900	−0.03	−0.08, 0.03	0.299
3–4 units	−0.01	−0.05, 0.03	0.741	−0.03	−0.08, 0.03	0.327
5+ units	0.00	−0.04, 0.05	0.849	−0.06	−0.11, −0.00	0.037
Mutually adjusted model						
<1 unit	0.02	−0.04, 0.09	0.478	−0.02	−0.09, 0.05	0.518
1–2 units	0.02	−0.03, 0.06	0.407	−0.04	−0.10, 0.02	0.179
3–4 units	0.02	−0.03, 0.06	0.448	−0.04	−0.10, 0.02	0.184
5+ units	0.04	−0.01, 0.08	0.116	−0.08	−0.14, −0.02	0.014
Fully and mutually adjusted model^∗^						
<1 unit	0.02	−0.04, 0.09	0.498	−0.01	−0.08, 0.06	0.813
1–2 units	0.01	−0.03, 0.05	0.625	−0.04	−0.10, 0.02	0.186
3–4 units	0.01	−0.04, 0.05	0.762	−0.03	−0.09, 0.03	0.270
5+ units	0.04	−0.01, 0.09	0.113	−0.05	−0.11, 0.01	0.079
In the first trimester of gestation						
Crude model						
<1 unit	0.03	−0.00, 0.06	0.055	−0.02	−0.08, 0.04	0.428
1–2 units	−0.02	−0.06, 0.02	0.272	−0.03	−0.08, 0.02	0.323
3–4 units	0.02	−0.04, 0.08	0.551	−0.03	−0.08, 0.02	0.263
5+ units	0.03	−0.04, 0.10	0.388	−0.05	−0.10, −0.00	0.035
Mutually adjusted model						
<1 unit	0.03	0.00, 0.06	0.046	−0.03	−0.09, 0.03	0.395
1–2 units	−0.02	−0.06, 0.02	0.318	−0.03	−0.08, 0.02	0.271
3–4 units	0.02	−0.03, 0.08	0.414	−0.03	−0.08, 0.02	0.209
5+ units	0.04	−0.03, 0.11	0.254	−0.06	−0.11, −0.01	0.024
Fully and mutually adjusted model^#^						
<1 unit	0.02	−0.00, 0.05	0.104	−0.02	−0.08, 0.04	0.488
1–2 units	−0.02	−0.05, 0.02	0.398	−0.03	−0.08, 0.02	0.174
3–4 units	0.04	−0.02, 0.10	0.151	−0.04	−0.09, 0.01	0.164
5+ units	0.05	−0.02, 0.12	0.151	−0.05	−0.10, 0.00	0.063

^∗^Test for trend maternal and paternal alcohol intake in full model: p = 0.113 and p = 0.084.

^#^Test for trend maternal and paternal alcohol intake in full model: p = 0.390 and p = 0.124.

**Table 3:** Maternal and Paternal Average Alcohol Dose per Occasion and Microcephaly at Birth – Odds Ratio (OR) and 95% Confidence Intervals (CI), MoBa data, Norway, 1999–2009.

	Mother			Father		
	OR	95% CI	*P*	OR	95% CI	*P*
Crude model						
Non drinker	Ref			Ref		
<1 unit	0.78	0.42, 1.47	0.450	1.41	0.70, 2.82	0.335
1–2 units	0.69	0.48, 0.99	0.046	1.10	0.60, 2.01	0.753
3–4 units	0.99	0.70, 1.41	0.963	1.30	0.71, 2.37	0.390
5+ units	0.92	0.63, 1.33	0.657	1.67	0.93, 3.00	0.086
Mutually adjusted model						
<1 unit	0.71	0.37, 1.35	0.298	1.69	0.82, 3.48	0.153
1–2 units	0.62	0.42, 0.92	0.017	1.39	0.73, 2.66	0.320
3–4 units	0.82	0.56, 1.21	0.321	1.61	0.84, 3.08	0.155
5+ units	0.70	0.47, 1.06	0.095	2.04	1.07, 3.89	0.031
Fully and mutually adjusted model^∗^						
<1 unit	0.67	0.35, 1.28	0.227	1.80	0.87, 3.72	0.111
1–2 units	0.63	0.42, 0.94	0.025	1.48	0.77, 2.84	0.238
3–4 units	0.80	0.54, 1.20	0.297	1.64	0.85, 3.16	0.140
5+ units	0.68	0.45, 1.04	0.078	1.93	1.01, 3.70	0.048
In the first trimester of gestation						
Crude model						
<1 unit	0.69	0.50, 0.94	0.019	0.90	0.48, 1.69	0.753
1–2 units	1.19	0.86, 1.66	0.294	0.99	0.60, 1.66	0.983
3–4 units	1.16	0.67, 1.98	0.600	1.13	0.68, 1.89	0.638
5+ units	1.41	0.79, 2.52	0.246	1.37	0.83, 2.27	0.220
Mutually adjusted model						
<1 unit	0.68	0.50, 0.93	0.017	0.92	0.49, 1.71	0.781
1–2 units	1.18	0.85, 1.65	0.329	1.02	0.61, 1.71	0.932
3–4 units	1.09	0.63, 1.87	0.755	1.16	0.69, 1.95	0.570
5+ units	1.29	0.72, 2.31	0.396	1.38	0.83, 2.29	0.211
Fully and mutually adjusted model^#^						
<1 unit	0.68	0.50, 0.94	0.019	1.00	0.53, 1.88	0.998
1–2 units	1.13	0.81, 1.59	0.470	1.11	0.66, 1.87	0.695
3–4 units	0.97	0.57, 1.68	0.925	1.21	0.72, 2.06	0.468
5+ units	1.22	0.68, 2.20	0.508	1.36	0.81, 2.28	0.246

^∗^Test for trend maternal and paternal alcohol intake in full model: p = 0.625 and p = 0.036.

^#^Test for trend maternal and paternal alcohol intake in full model: p = 0.545 and p = 0.056.

**Table 4:** Maternal and Paternal Average Alcohol Dose per Occasion and Microcephaly at 3 Months Post-Partum – Odds Ratio (OR) and 95% Confidence Intervals (CI), MoBa data, Norway, 1999–2009.

	Mother			Father		
	OR	95% CI	*P*	OR	95% CI	*P*
Crude model						
Non drinker	Ref			Ref		
<1 unit	1.10	0.71, 1.72	0.660	1.51	0.91, 2.48	0.108
1–2 units	1.01	0.77, 1.34	0.929	1.20	0.78, 1.85	0.413
3–4 units	1.09	0.82, 1.44	0.550	1.41	0.91, 2.17	0.122
5+ units	1.02	0.77, 1.37	0.877	1.49	0.97, 2.28	0.068
Mutually adjusted model						
<1 unit	1.03	0.65, 1.62	0.915	1.56	0.93, 2.62	0.094
1–2 units	0.93	0.69, 1.26	0.655	1.25	0.78, 2.00	0.349
3–4 units	0.96	0.70, 1.30	0.774	1.48	0.92, 2.37	0.104
5+ units	0.86	0.62, 1.19	0.372	1.60	1.00, 2.56	0.051
Fully and mutually adjusted model^∗^						
<1 unit	1.02	0.64, 1.60	0.948	1.59	0.94, 2.68	0.083
1–2 units	1.00	0.73, 1.36	0.984	1.26	0.79, 2.02	0.338
3–4 units	1.01	0.74, 1.37	0.973	1.47	0.92, 2.37	0.109
5+ units	0.87	0.63, 1.21	0.412	1.52	0.95, 2.44	0.081
In the first trimester of gestation						
Crude model						
<1 unit	0.82	0.66, 1.01	0.063	1.14	0.73, 1.78	0.563
1–2 units	0.88	0.67, 1.15	0.344	1.07	0.73, 1.56	0.733
3–4 units	1.20	0.82, 1.76	0.358	1.28	0.88, 1.87	0.202
5+ units	0.91	0.54, 1.52	0.709	1.29	0.89, 1.88	0.177
Mutually adjusted model						
<1 unit	0.81	0.66, 1.00	0.051	1.16	0.74, 1.81	0.514
1–2 units	0.86	0.66, 1.14	0.294	1.10	0.76, 1.61	0.608
3–4 units	1.15	0.78, 1.69	0.472	1.33	0.91, 1.94	0.145
5+ units	0.86	0.51, 1.45	0.577	1.33	0.91, 1.94	0.138
Fully and mutually adjusted model^#^						
<1 unit	0.82	0.66, 1.02	0.071	1.25	0.79, 1.95	0.338
1–2 units	0.82	0.62, 1.09	0.171	1.16	0.79, 1.71	0.439
3–4 units	1.08	0.73, 1.59	0.708	1.38	0.94, 2.03	0.102
5+ units	0.82	0.49, 1.39	0.461	1.33	0.90, 1.95	0.148

^∗^Test for trend maternal and paternal alcohol intake in full model: p = 0.089 and p = 0.063.

^#^Test for trend maternal and paternal alcohol intake in full model: p = 0.178 and p = 0.090.
