# Erratum: Pre-conception and prenatal alcohol exposure from mothers and fathers drinking and head circumference: results from the Norwegian Mother-Child Study (MoBa)

**DOI:** 10.1038/srep45877

**Published:** 2017-04-24

**Authors:** Luisa Zuccolo, Lisa A. DeRoo, Andrew K. Wills, George Davey Smith, Pål Suren, Christine Roth, Camilla Stoltenberg, Per Magnus

Scientific Reports 6: Article number: 3953510.1038/srep39535; published online 12 23 2016; updated on 04 24 2017

The original HTML version of this Article listed an incorrect volume number. This has now been corrected in the HTML version; the PDF version was correct at the time of publication.

