# Supplementary material for: Pre-conception and prenatal alcohol exposure from mothers and fathers drinking and head circumference: results from the Norwegian Mother-Child Study (MoBa)
Source: Sci Rep. 2016 Dec 23;6:39535. doi: 10.1038/srep39535 (PMC5180191; doi:10.1038/srep39535)
Supplement: Supplementary Information [file srep39535-s1.pdf]

Supplementary Information

Pre-conception and prenatal alcohol exposure from mothers and fathers drinking and head circumference: results from the Norwegian Mother-Child Study (MoBa).

Luisa Zuccolo[1]; Lisa A. DeRoo[2]; Andrew K Wills[3]; George Davey Smith[1]; Pål Suren[4]; Christine Roth[4,5]; Camilla Stoltenberg[6]; Per Magnus[4]

Table of contents

Description of confounders-alcohol associations 2

Description of confounders-outcomes associations 3

TABLES AND FIGURES 4

Alcohol drinking patterns 4

Alcohol-Confounders associations 6

Confounders-Outcomes associations 20

Sensitivity analyses 22

Description of confounders-alcohol associations

Confounders-alcohol associations were similar or very similar for mothers and fathers (with a few exceptions), with maternal characteristics more strongly associated with maternal drinking and vice versa, and directions of association as expected (Supplementary Information, Tables S7-S10, Figures S1 and S2).

For maternal drinking before pregnancy, the shape of association was often curvilinear (U, J or inverse U and J), so often non-drinkers and heavier drinkers were more like each other, and differed from light-to-moderate drinkers (drinking 1-2 drinks per occasion), in particular in terms of socioeconomic factors (Supplementary Information, Table S7). The latter was the

category most likely to be taking folic acid supplements, to have planned the pregnancy, to be recruited earlier, to have taller partners with lower BMI, and both themselves and their partners were more likely to be older, better educated, and earning more. Where confounders-alcohol relationship was monotonic, increasing alcohol use was associated with no previous pregnancies, Norwegian ethnic origin, no financial strains, increased smoking and drug taking in pregnancy (maternal and paternal).

Paternal drinking before pregnancy was less obviously associated with maternal pre-pregnancy BMI and gross income, or paternal height, compared to maternal drinking (Supplementary Information, Tables S7 and S8, Figure S1).

Most women quit drinking in the first trimester of pregnancy or markedly reduced their alcohol intake (Supplementary Information, Tables S4 and S9). The biggest differences compared to before pregnancy were in the patterning of maternal drinking in association with planned pregnancy, which went from U-shaped to monotonic), and with maternal smoking in pregnancy, folic acid and financial strain, which went from showing some to no association (Supplementary Information, Tables S7 and S9, Figures S1 and S2).

Paternal drinking patterns of association were very stable before-during pregnancy

(Supplementary Information, Tables S8 Vs S10). The patterning of alcohol drinking duringpregnancy in association with most confounders was very similar for mothers and fathers, with the exception of maternal ethnic background and education, and maternal and paternal age and smoking during pregnancy, and paternal age (Supplementary Information,F igure S2).

Description of confounders-outcomes associations

There was evidence of association with offspring head circumference at birth for most of the potential confounders identified, the only exceptions being financial strain and planned pregnancy (Supplementary Information,Tables S11 Vs S12). The strength and direction of associations were similar for the outcome at 3 months, except for maternal and paternal education and gross income, which went from an inverse U shape, with babies of parents at both ends of the education and income spectrum having smaller head circumferences compared to babies born to parents in the middle of the spectrum, to a positive trend, with increasing head size for increasing education levels and wealth (Supplementary Information,Tables S11 Vs S12), possibly suggesting catch-up growth for children of richer, better educated parents. The other difference was for folic acid use, which went from a negative association with head circumference at birth to a borderline positive association at 3 months. Breech fetal presentation predicts larger head circumference at birth but the correlation is less strong for head circumference measured 3 months, which was the motivation for repeating all analyses with the outcome measured at 3 months, to overcome at least some of the bias potentially caused by this association.

TABLES AND FIGURES

Alcohol drinking patterns

Supplementary Information, Table S1. Maternal Vs paternal alcohol intake before pregnancy (units per occasion) – correlation coefficients.

	0	<1	1-2	3-4	5+
0	0.448	0.025	-0.071	-0.106	-0.102
<1	0.091	0.075	0.032	-0.054	-0.064
1-2	-0.014	0.048	0.235	-0.052	-0.205
3-4	-0.079	-0.021	0.055	0.095	-0.101
5+	-0.138	-0.070	-0.253	0.030	0.352

Supplementary Information, Table S2. Maternal Vs paternal alcohol intake during 1st trimester of pregnancy (units per occasion) – correlation coefficients.

	0	<1	1-2	3-4	5+
0	0.111	-0.072	-0.052	-0.033	-0.028
<1	0.062	-0.036	-0.031	-0.022	-0.017
1-2	-0.005	0.035	0.019	-0.034	-0.062
3-4	-0.052	0.046	0.035	0.006	-0.025
5+	-0.031	-0.025	-0.011	0.055	0.106

Supplementary Information, Table S3. Paternal alcohol intake before Vs during 1st trimester of pregnancy (units per occasion) – correlation coefficients.

	0	<1	1-2	3-4	5+
0	0.895	0.052	-0.123	-0.126	-0.157
<1	-0.044	0.764	-0.052	-0.123	-0.173
1-2	-0.134	-0.149	0.859	-0.241	-0.455
3-4	-0.118	-0.135	-0.356	0.828	-0.311
5+	-0.141	-0.162	-0.430	-0.408	0.900

Supplementary Information, Table S4. Maternal alcohol intake before Vs during 1st trimester of pregnancy (units per occasion) – correlation coefficients.

	0	<1	1-2	3-4	5+
0	0.184	0.074	0.021	-0.099	-0.063
<1	-0.120	-0.024	0.052	0.060	-0.034
1-2	-0.088	-0.053	0.024	0.080	-0.034
3-4	-0.052	-0.033	-0.111	0.083	0.076
5+	-0.047	-0.029	-0.103	-0.086	0.241

Supplementary Information, Table S5. Maternal alcohol intake during 1st Vs 2nd trimester of pregnancy (units per occasion) -– correlation coefficients.

	0	<1	1-2	3-4	5+
0	0.431	-0.343	-0.237	-0.028	0.001
<1	-0.365	0.393	0.085	0.004	-0.012
1-2	-0.208	-0.006	0.316	0.040	0.012
3-4	-0.027	-0.008	0.009	0.053	0.022
5+	-0.014	-0.006	-0.004	0.013	0.049

Supplementary Information, Table S6. Maternal alcohol intake during 2nd Vs 3rd trimester of pregnancy (units per occasion) -– correlation coefficients.

	0	<1	1-2	3-4	5+
0	0.716	-0.610	-0.343	-0.032	-0.014
<1	-0.525	0.603	-0.011	-0.007	-0.004
1-2	-0.443	0.171	0.571	0.013	-0.003
3-4	-0.051	0.023	0.021	0.260	0.034
5+	-0.013	-0.003	0.012	0.029	0.203

Alcohol-Confounders associations

Supplementary Information, Table S7. Association between confounders and maternal alcohol before pregnancy (in units/occasion) – numbers (%).

	Total	0 units/occasi	<1	1-2	3-4	5+
Year of birth						
1999-2003	12593	1094 (8.69)	428 (3.40)	3727 (29.60)	4024 (31.95)	3320 (26.36)
2004-2005	20687	1751 (8.46)	710 (3.43)	6377 (30.83)	6317 (30.54)	5532 (26.74)
2006-2007	23196	1781 (7.68)	772 (3.33)	7492 (32.30)	7200 (31.04)	5951 (25.66)
2008-2009	11622	833 (7.17)	368 (3.17)	3984 (34.28)	3600 (30.98)	2837 (24.41)
Fetal presentation						
Normal cephalic	61872	4993 (8.07)	2090 (3.38)	19720 (31.87)	19130 (30.92)	15939 (25.76)
Breech	2503	167 (6.67)	75 (3.00)	722 (28.85)	859 (34.32)	680 (27.17)
Transverse	152	20 (13.16)	1 (0.66)	64 (42.11)	41 (26.97)	26 (17.11)
Anomaly cephalic	3026	233 (7.70)	95 (3.14)	884 (29.21)	944 (31.20)	870 (28.75)
Birth weight (g)						
<2500	1334	120 (9.00)	50 (3.75)	344 (25.79)	439 (32.91)	381 (28.56)
2500-2999	5882	480 (8.16)	201 (3.42)	1729 (29.39)	1918 (32.61)	1554 (26.42)
3000-3499	20035	1598 (7.98)	664 (3.31)	6351 (31.70)	6285 (31.37)	5137 (25.64)
3500-3999	25758	2012 (7.81)	862 (3.35)	8275 (32.13)	7940 (30.83)	6669 (25.89)
4000+	15089	1249 (8.28)	501 (3.32)	4881 (32.35)	4559 (30.21)	3899 (25.84)
Folic acid use around conception						
No	16126	1605 (9.95)	612 (3.80)	4401 (27.29)	4726 (29.31)	4782 (29.65)
Yes	49852	3622 (7.27)	1577 (3.16)	16609 (33.32)	15785 (31.66)	12259 (24.59)
Planned pregnancy						
No	11621	1043 (8.98)	406 (3.49)	2963 (25.50)	3242 (27.90)	3967 (34.14)
Yes	55734	4326 (7.76)	1839 (3.30)	18410 (33.03)	17682 (31.73)	13477 (24.18)
Parity						
0	32191	1932 (6.00)	827 (2.57)	8003 (24.86)	10657 (33.11)	10772 (33.46)
1+	34998	3438 (9.82)	1420 (4.06)	13333 (38.10)	10217 (29.19)	6590 (18.83)
Ethnic background						
Norwegian	56229	4057 (7.22)	1681 (2.99)	17401 (30.95)	17713 (31.50)	15377 (27.35)
Other	10599	1308 (12.34)	544 (5.13)	3844 (36.27)	3047 (28.75)	1856 (17.51)
Financial strain						
Yes	39280	3404 (8.67)	1349 (3.43)	12937 (32.94)	12163 (30.96)	9427 (24.00)
No	27597	1899 (6.88)	869 (3.15)	8283 (30.01)	8642 (31.31)	7904 (28.64)
Maternal age (years)						
<25	8285	807 (9.74)	278 (3.36)	1197 (14.45)	1794 (21.65)	4209 (50.80)
25-29	24270	2012 (8.29)	703 (2.90)	6267 (25.82)	7554 (31.12)	7734 (31.87)
30-34	25626	1837 (7.17)	850 (3.32)	9722 (37.94)	8579 (33.48)	4638 (18.10)
35+	9917	803 (8.10)	447 (4.51)	4394 (44.31)	3214 (32.41)	1059 (10.68)
Maternal height (cm)						
<164	14874	1576 (10.60)	595 (4.00)	4701 (31.61)	4281 (28.78)	3721 (25.02)
164-167	14526	1179 (8.12)	504 (3.47)	4653 (32.03)	4460 (30.70)	3730 (25.68)
168-171	18317	1347 (7.35)	536 (2.93)	5724 (31.25)	5845 (31.91)	4865 (26.56)
172+	19662	1282 (6.52)	606 (3.08)	6291 (32.00)	6353 (32.31)	5130 (26.09)
Maternal pre-preg BMI (kg/m2)						
<20	8426	775 (9.20)	355 (4.21)	3033 (36.00)	2414 (28.65)	1849 (21.94)
20-24	37171	2728 (7.34)	1142 (3.07)	12233 (32.91)	12048 (32.41)	9020 (24.27)
25-29	14437	1199 (8.31)	447 (3.10)	4075 (28.23)	4392 (30.42)	4324 (29.95)
30-35	4648	404 (8.69)	177 (3.81)	1273 (27.39)	1372 (29.52)	1422 (30.59)
35+	1669	178 (10.67)	79 (4.73)	435 (26.06)	436 (26.12)	541 (32.41)
Maternal gross income						
<150k	11218	1548 (13.80)	515 (4.59)	2843 (25.34)	2729 (24.33)	3583 (31.94)
150-199K	6782	735 (10.84)	247 (3.64)	1835 (27.06)	1863 (27.47)	2102 (30.99)
200-299k	22183	1698 (7.65)	745 (3.36)	6769 (30.51)	6750 (30.43)	6221 (28.04)
300-399k	17525	862 (4.92)	450 (2.57)	6304 (35.97)	6162 (35.16)	3747 (21.38)
400k+	8144	315 (3.87)	203 (2.49)	3164 (38.85)	3042 (37.35)	1420 (17.44)
Maternal education						
High school (not completed)	4770	670 (14.05)	217 (4.55)	958 (20.08)	1097 (23.00)	1828 (38.32)
High school (completed)	17744	1528 (8.61)	670 (3.78)	3927 (22.13)	4870 (27.45)	6749 (38.04)
College level	27941	2203 (7.88)	809 (2.90)	9177 (32.84)	9214 (32.98)	6538 (23.40)
Postgraduate level	16226	931 (5.74)	533 (3.28)	7120 (43.88)	5511 (33.96)	2131 (13.13)
Maternal smoking in pregnancy (cigs/day)						

Non smoker	64265	5242 (8.16)	2179 (3.39)	20915 (32.54)	20125 (31.32)	15804 (24.59)
<2	1711	79 (4.62)	38 (2.22)	298 (17.42)	495 (28.93)	801 (46.81)
2-4	452	21 (4.65)	10 (2.21)	62 (13.72)	117 (25.88)	242 (53.54)
4+	1266	82 (6.48)	32 (2.53)	182 (14.38)	286 (22.59)	684 (54.03)
Maternal drugs use in pregnancy						
No	56991	4827 (8.47)	2030 (3.56)	18925 (33.21)	17550 (30.79)	13659 (23.97)
Yes	109	6 (5.50)	3 (2.75)	10 (9.17)	21 (19.27)	69 (63.30)
Paternal age (years)						
<25	3103	278 (8.96)	108 (3.48)	419 (13.50)	620 (19.98)	1678 (54.08)
25-29	15543	1324 (8.52)	442 (2.84)	3339 (21.48)	4496 (28.93)	5942 (38.23)
30-34	27108	1938 (7.15)	834 (3.08)	8787 (32.41)	8914 (32.88)	6635 (24.48)
35+	22322	1917 (8.59)	893 (4.00)	9031 (40.46)	7106 (31.83)	3375 (15.12)
Paternal height (cm)						
<178	16269	1573 (9.67)	561 (3.45)	4857 (29.85)	4848 (29.80)	4430 (27.23)
178-180	14756	1171 (7.94)	510 (3.46)	4788 (32.45)	4548 (30.82)	3739 (25.34)
181-185	18224	1308 (7.18)	609 (3.34)	5862 (32.17)	5687 (31.21)	4758 (26.11)
186+	17763	1297 (7.30)	560 (3.15)	5771 (32.49)	5738 (32.30)	4397 (24.75)
Paternal BMI (kg/m2)						
<25	29262	2376 (8.12)	937 (3.20)	9858 (33.69)	9003 (30.77)	7088 (24.22)
25-29	30208	2247 (7.44)	992 (3.28)	9353 (30.96)	9594 (31.76)	8022 (26.56)
30-35	5819	539 (9.26)	233 (4.00)	1600 (27.50)	1742 (29.94)	1705 (29.30)
35+	976	114 (11.68)	55 (5.64)	238 (24.39)	263 (26.95)	306 (31.35)
Paternal gross income						
<150k	4227	560 (13.25)	178 (4.21)	1027 (24.30)	1093 (25.86)	1369 (32.39)
150-199K	2579	328 (12.72)	106 (4.11)	653 (25.32)	691 (26.79)	801 (31.06)
200-299k	13446	1323 (9.84)	483 (3.59)	3527 (26.23)	3882 (28.87)	4231 (31.47)
300-399k	21802	1623 (7.44)	696 (3.19)	6862 (31.47)	6761 (31.01)	5860 (26.88)
400k+	23157	1257 (5.43)	706 (3.05)	8730 (37.70)	7932 (34.25)	4532 (19.57)
Paternal education						
High school (not completed)	6745	743 (11.02)	275 (4.08)	1470 (21.79)	1715 (25.43)	2542 (37.69)
High school (completed)	25489	2101 (8.24)	858 (3.37)	6244 (24.50)	7309 (28.68)	8977 (35.22)
College level	18593	1456 (7.83)	568 (3.05)	6424 (34.55)	6330 (34.05)	3815 (20.52)
Postgraduate level	16444	1075 (6.54)	545 (3.31)	7199 (43.78)	5545 (33.72)	2080 (12.65)
Paternal smoking in pregnancy (cigs/day)						
Non smoker	51795	4434 (8.56)	1841 (3.55)	17857 (34.48)	16167 (31.21)	11496 (22.20)
<5	7327	380 (5.19)	162 (2.21)	1866 (25.47)	2468 (33.68)	2451 (33.45)
5-10	1122	79 (7.04)	40 (3.57)	273 (24.33)	303 (27.01)	427 (38.06)
10+	6956	476 (6.84)	205 (2.95)	1368 (19.67)	1923 (27.65)	2984 (42.90)
Paternal drugs use in pregnancy						
No	54621	4889 (8.95)	1991 (3.65)	18105 (33.15)	16596 (30.38)	13040 (23.87)
Yes	360	7 (1.94)	2 (0.56)	53 (14.72)	136 (37.78)	162 (45.00)

Supplementary Information, Table S8. Association between confounders and paternal alcohol before pregnancy (in units/occasion) – numbers (%).

	Total	0	<1	1-2	3-4	5+
Year of birth						
1999-2003	12593	532 (4.22)	219 (1.74)	3617 (28.72)	3497 (27.77)	4728 (37.54)
2004-2005	20687	856 (4.14)	299 (1.45)	6356 (30.72)	5569 (26.92)	7607 (36.77)
2006-2007	23196	891 (3.84)	1945 (8.39)	5988 (25.81)	5787 (24.95)	8585 (37.01)
2008-2009	11622	424 (3.65)	1072 (9.22)	2938 (25.28)	2792 (24.02)	4396 (37.82)
Fetal presentation						
Normal cephalic	61872	2463 (3.98)	3246 (5.25)	17270 (27.91)	15993 (25.85)	22900 (37.01)
Breech	2503	76 (3.04)	129 (5.15)	644 (25.73)	674 (26.93)	980 (39.15)
Transverse	152	7 (4.61)	10 (6.58)	44 (28.95)	40 (26.32)	51 (33.55)
Anomaly cephalic	3026	136 (4.49)	135 (4.46)	758 (25.05)	805 (26.60)	1192 (39.39)
Birth weight (g)						
<2500	1334	46 (3.45)	67 (5.02)	331 (24.81)	312 (23.39)	578 (43.33)
2500-2999	5882	250 (4.25)	317 (5.39)	1560 (26.52)	1426 (24.24)	2329 (39.60)
3000-3499	20035	784 (3.91)	1033 (5.16)	5477 (27.34)	5212 (26.01)	7529 (37.58)
3500-3999	25758	1000 (3.88)	1360 (5.28)	7158 (27.79)	6667 (25.88)	9573 (37.17)
4000+	15089	623 (4.13)	758 (5.02)	4373 (28.98)	4028 (26.69)	5307 (35.17)
Folic acid use around conception						
No	16126	778 (4.82)	686 (4.25)	4294 (26.63)	4049 (25.11)	6319 (39.19)
Yes	49852	1812 (3.63)	2747 (5.51)	14012 (28.11)	13065 (26.21)	18216 (36.54)
Planned pregnancy						
No	11621	479 (4.12)	612 (5.27)	2849 (24.52)	2615 (22.50)	5066 (43.59)
Yes	55734	2177 (3.91)	2884 (5.17)	15845 (28.43)	14839 (26.62)	19989 (35.87)
Parity						
0	32191	1132 (3.52)	1339 (4.16)	6934 (21.54)	7909 (24.57)	14877 (46.21)
1+	34998	1523 (4.35)	2157 (6.16)	11743 (33.55)	9510 (27.17)	10065 (28.76)
Ethnic background						
Norwegian	56229	1954 (3.48)	2764 (4.92)	15252 (27.12)	14627 (26.01)	21632 (38.47)
Other	10599	699 (6.59)	710 (6.70)	3321 (31.33)	2703 (25.50)	3166 (29.87)
Financial strain						
Yes	39280	1650 (4.20)	2002 (5.10)	11235 (28.60)	10348 (26.34)	14045 (35.76)
No	27597	964 (3.49)	1460 (5.29)	7324 (26.54)	7009 (25.40)	10840 (39.28)
Maternal age (years)						
<25	8285	441 (5.32)	404 (4.88)	1352 (16.32)	1466 (17.69)	4622 (55.79)
25-29	24270	1028 (4.24)	1189 (4.90)	5817 (23.97)	5863 (24.16)	10373 (42.74)
30-34	25626	843 (3.29)	1321 (5.15)	8147 (31.79)	7392 (28.85)	7923 (30.92)
35+	9917	391 (3.94)	621 (6.26)	3583 (36.13)	2924 (29.48)	2398 (24.18)
Maternal height (cm)						
<164	14874	726 (4.88)	837 (5.63)	4130 (27.77)	3676 (24.71)	5505 (37.01)
164-167	14526	548 (3.77)	837 (5.76)	4001 (27.54)	3728 (25.66)	5412 (37.26)
168-171	18317	714 (3.90)	890 (4.86)	4998 (27.29)	4829 (26.36)	6886 (37.59)
172+	19662	670 (3.41)	942 (4.79)	5569 (28.32)	5222 (26.56)	7259 (36.92)
Maternal pre-preg BMI (kg/m2)						
<20	8426	357 (4.24)	470 (5.58)	2347 (27.85)	2128 (25.26)	3124 (37.08)
20-24	37171	1394 (3.75)	1782 (4.79)	10336 (27.81)	9798 (26.36)	13861 (37.29)
25-29	14437	545 (3.78)	772 (5.35)	3978 (27.55)	3731 (25.84)	5411 (37.48)
30-35	4648	224 (4.82)	292 (6.28)	1285 (27.65)	1177 (25.32)	1670 (35.93)
35+	1669	81 (4.85)	138 (8.27)	456 (27.32)	376 (22.53)	618 (37.03)
Maternal gross income						
<150k	11218	765 (6.82)	647 (5.77)	2788 (24.85)	2330 (20.77)	4688 (41.79)
150-199K	6782	367 (5.41)	364 (5.37)	1808 (26.66)	1585 (23.37)	2658 (39.19)
200-299k	22183	834 (3.76)	1064 (4.80)	6280 (28.31)	5686 (25.63)	8319 (37.50)
300-399k	17525	426 (2.43)	961 (5.48)	5000 (28.53)	5010 (28.59)	6128 (34.97)
400k+	8144	150 (1.84)	361 (4.43)	2392 (29.37)	2508 (30.80)	2733 (33.56)
Maternal education						
High school (not completed)	4770	287 (6.02)	237 (4.97)	1043 (21.87)	982 (20.59)	2221 (46.56)
High school (completed)	17744	684 (3.85)	948 (5.34)	4137 (23.31)	4112 (23.17)	7863 (44.31)
College level	27941	1169 (4.18)	1479 (5.29)	7956 (28.47)	7360 (26.34)	9977 (35.71)
Postgraduate level	16226	486 (3.00)	805 (4.96)	5389 (33.21)	4823 (29.72)	4723 (29.11)
Maternal smoking in pregnancy (cigs/day)						
Non smoker	64265	2626 (4.09)	3376 (5.25)	18126 (28.21)	16802 (26.14)	23335 (36.31)
<2	1711	32 (1.87)	73 (4.27)	344 (20.11)	366 (21.39)	896 (52.37)

2-4	452	5 (1.11)	11 (2.43)	86 (19.03)	99 (21.90)	251 (55.53)
4+	1266	24 (1.90)	54 (4.27)	242 (19.12)	276 (21.80)	670 (52.92)
Maternal drugs use in pregnancy						
No	56991	2377 (4.17)	3100 (5.44)	16476 (28.91)	14915 (26.17)	20123 (35.31)
Yes	109	1 (0.92)	8 (7.34)	14 (12.84)	13 (11.93)	73 (66.97)
Paternal age (years)						
<25	3103	147 (4.74)	149 (4.80)	445 (14.34)	446 (14.37)	1916 (61.75)
25-29	15543	720 (4.63)	748 (4.81)	3221 (20.72)	3393 (21.83)	7461 (48.00)
30-34	27108	948 (3.50)	1276 (4.71)	7636 (28.17)	7321 (27.01)	9927 (36.62)
35+	22322	888 (3.98)	1358 (6.08)	7593 (34.02)	6477 (29.02)	6006 (26.91)
Paternal height (cm)						
<178	16269	787 (4.84)	928 (5.70)	4539 (27.90)	4160 (25.57)	5855 (35.99)
178-180	14756	575 (3.90)	771 (5.22)	4158 (28.18)	3815 (25.85)	5437 (36.85)
181-185	18224	618 (3.39)	957 (5.25)	5064 (27.79)	4687 (25.72)	6898 (37.85)
186+	17763	663 (3.73)	853 (4.80)	4820 (27.14)	4702 (26.47)	6725 (37.86)
Paternal BMI (kg/m2)						
<25	29262	1270 (4.34)	1503 (5.14)	8848 (30.24)	7568 (25.86)	10073 (34.42)
25-29	30208	1040 (3.44)	1538 (5.09)	7890 (26.12)	8069 (26.71)	11671 (38.64)
30-35	5819	246 (4.23)	357 (6.14)	1385 (23.80)	1333 (22.91)	2498 (42.93)
35+	976	45 (4.61)	89 (9.12)	226 (23.16)	203 (20.80)	413 (42.32)
Paternal gross income						
<150k	4227	382 (9.04)	269 (6.36)	963 (22.78)	807 (19.09)	1806 (42.73)
150-199K	2579	197 (7.64)	160 (6.20)	632 (24.51)	559 (21.68)	1031 (39.98)
200-299k	13446	683 (5.08)	660 (4.91)	3557 (26.45)	3097 (23.03)	5449 (40.53)
300-399k	21802	745 (3.42)	1233 (5.66)	6111 (28.03)	5635 (25.85)	8078 (37.05)
400k+	23157	491 (2.12)	1126 (4.86)	6838 (29.53)	6846 (29.56)	7856 (33.92)
Paternal education						
High school (not completed)	6745	348 (5.16)	399 (5.92)	1528 (22.65)	1434 (21.26)	3036 (45.01)
High school (completed)	25489	995 (3.90)	1339 (5.25)	6102 (23.94)	5950 (23.34)	11103 (43.56)
College level	18593	786 (4.23)	926 (4.98)	5491 (29.53)	5100 (27.43)	6290 (33.83)
Postgraduate level	16444	525 (3.19)	830 (5.05)	5570 (33.87)	4930 (29.98)	4589 (27.91)
Paternal smoking in pregnancy (cigs/day)						
Non smoker	51795	2365 (4.57)	2926 (5.65)	15670 (30.25)	13872 (26.78)	16962 (32.75)
<5	7327	113 (1.54)	226 (3.08)	1472 (20.09)	1820 (24.84)	3696 (50.44)
5-10	1122	30 (2.67)	59 (5.26)	218 (19.43)	258 (22.99)	557 (49.64)
10+	6956	148 (2.13)	278 (4.00)	1337 (19.22)	1480 (21.28)	3713 (53.38)
Paternal drugs use in pregnancy						
No	54621	2491 (4.56)	3095 (5.67)	16228 (29.71)	14472 (26.50)	18335 (33.57)
Yes	360	2 (0.56)	8 (2.22)	35 (9.72)	62 (17.22)	253 (70.28)

Supplementary Information, Table S9. Association between confounders and maternal alcohol in 1st trimester pregnancy (in units/occasion) – numbers (%)

	Total	0	<1	1-2	3-4	5+
Year of birth						
1999-2003	12593	8221 (65.28)	2347 (18.64)	1312 (10.42)	400 (3.18)	313 (2.49)
2004-2005	20687	14170 (68.50)	3518 (17.01)	1837 (8.88)	650 (3.14)	512 (2.47)
2006-2007	23196	16831 (72.56)	3426 (14.77)	1671 (7.20)	708 (3.05)	560 (2.41)
2008-2009	11622	8550 (73.57)	1613 (13.88)	797 (6.86)	369 (3.18)	293 (2.52)
Fetal presentation						
Normal cephalic	61872	43514 (70.33)	9863 (15.94)	5098 (8.24)	1905 (3.08)	1492 (2.41)
Breech	2503	1741 (69.56)	405 (16.18)	192 (7.67)	98 (3.92)	67 (2.68)
Transverse	152	105 (69.08)	26 (17.11)	16 (10.53)	4 (2.63)	1 (0.66)
Anomaly cephalic	3026	2051 (67.78)	518 (17.12)	255 (8.43)	96 (3.17)	106 (3.50)
Birth weight (g)						
<2500	1334	955 (71.59)	177 (13.27)	102 (7.65)	58 (4.35)	42 (3.15)
2500-2999	5882	4161 (70.74)	916 (15.57)	481 (8.18)	180 (3.06)	144 (2.45)
3000-3499	20035	14008 (69.92)	3221 (16.08)	1692 (8.45)	636 (3.17)	478 (2.39)
3500-3999	25758	18037 (70.02)	4166 (16.17)	2121 (8.23)	779 (3.02)	655 (2.54)
4000+	15089	10611 (70.32)	2424 (16.06)	1221 (8.09)	474 (3.14)	359 (2.38)
Folic acid use around conception						
No	16126	11074 (68.67)	2500 (15.50)	1502 (9.31)	545 (3.38)	505 (3.13)
Yes	49852	35181 (70.57)	8091 (16.23)	3951 (7.93)	1514 (3.04)	1115 (2.24)
Planned pregnancy						
No	11621	7381 (63.51)	1948 (16.76)	1187 (10.21)	576 (4.96)	529 (4.55)
Yes	55734	39870 (71.54)	8848 (15.88)	4354 (7.81)	1533 (2.75)	1129 (2.03)
Parity						
0	32191	21979 (68.28)	5096 (15.83)	2618 (8.13)	1355 (4.21)	1143 (3.55)
1+	34998	25156 (71.88)	5670 (16.20)	2912 (8.32)	746 (2.13)	514 (1.47)
Ethnic background						
Norwegian	56229	39711 (70.62)	8765 (15.59)	4522 (8.04)	1786 (3.18)	1445 (2.57)
Other	10599	7165 (67.60)	1947 (18.37)	1002 (9.45)	297 (2.80)	188 (1.77)
Financial strain						
Yes	39280	27452 (69.89)	6484 (16.51)	3243 (8.26)	1192 (3.03)	909 (2.31)
No	27597	19420 (70.37)	4230 (15.33)	2289 (8.29)	906 (3.28)	752 (2.72)
Maternal age (years)						
<25	8285	6576 (79.37)	791 (9.55)	321 (3.87)	234 (2.82)	363 (4.38)
25-29	24270	17629 (72.64)	3406 (14.03)	1685 (6.94)	807 (3.33)	743 (3.06)
30-34	25626	17358 (67.74)	4637 (18.09)	2362 (9.22)	804 (3.14)	465 (1.81)
35+	9917	6209 (62.61)	2070 (20.87)	1249 (12.59)	282 (2.84)	107 (1.08)
Maternal height (cm)						
<164	14874	10826 (72.78)	2123 (14.27)	1175 (7.90)	435 (2.92)	315 (2.12)
164-167	14526	10314 (71.00)	2243 (15.44)	1158 (7.97)	455 (3.13)	356 (2.45)
168-171	18317	12645 (69.03)	3064 (16.73)	1553 (8.48)	589 (3.22)	466 (2.54)
172+	19662	13480 (68.56)	3369 (17.13)	1668 (8.48)	628 (3.19)	517 (2.63)
Maternal pre-preg BMI (kg/m2)						
<20	8426	5951 (70.63)	1345 (15.96)	696 (8.26)	251 (2.98)	183 (2.17)
20-24	37171	25331 (68.15)	6433 (17.31)	3246 (8.73)	1239 (3.33)	922 (2.48)
25-29	14437	10424 (72.20)	2094 (14.50)	1111 (7.70)	430 (2.98)	378 (2.62)
30-35	4648	3506 (75.43)	596 (12.82)	328 (7.06)	114 (2.45)	104 (2.24)
35+	1669	1308 (78.37)	189 (11.32)	92 (5.51)	40 (2.40)	40 (2.40)
Maternal gross income						
<150k	11218	8485 (75.64)	1407 (12.54)	677 (6.03)	306 (2.73)	343 (3.06)
150-199K	6782	5072 (74.79)	876 (12.92)	467 (6.89)	199 (2.93)	168 (2.48)
200-299k	22183	15902 (71.69)	3263 (14.71)	1781 (8.03)	690 (3.11)	547 (2.47)
300-399k	17525	11742 (67.00)	3164 (18.05)	1599 (9.12)	591 (3.37)	429 (2.45)
400k+	8144	4916 (60.36)	1865 (22.90)	922 (11.32)	296 (3.63)	145 (1.78)
Maternal education						
High school (not completed)	4770	3737 (78.34)	512 (10.73)	272 (5.70)	100 (2.10)	149 (3.12)
High school (completed)	17744	13245 (74.64)	2178 (12.27)	1198 (6.75)	541 (3.05)	582 (3.28)
College level	27941	19481 (69.72)	4475 (16.02)	2357 (8.44)	942 (3.37)	686 (2.46)
Postgraduate level	16226	10344 (63.75)	3506 (21.61)	1656 (10.21)	500 (3.08)	220 (1.36)
Maternal smoking in pregnancy (cigs/day)						
Non smoker	64265	45108 (70.19)	10398 (16.18)	5242 (8.16)	1990 (3.10)	1527 (2.38)
<2	1711	1181 (69.02)	243 (14.20)	149 (8.71)	64 (3.74)	74 (4.32)

2-4	452	334 (73.89)	52 (11.50)	42 (9.29)	14 (3.10)	10 (2.21)
4+	1266	877 (69.27)	151 (11.93)	144 (11.37)	41 (3.24)	53 (4.19)
Maternal drugs use in pregnancy						
No	56991	40968 (71.89)	8797 (15.44)	4451 (7.81)	1612 (2.83)	1163 (2.04)
Yes	109	37 (33.94)	24 (22.02)	21 (19.27)	10 (9.17)	17 (15.60)
Paternal age (years)						
<25	3103	2432 (78.38)	297 (9.57)	126 (4.06)	95 (3.06)	153 (4.93)
25-29	15543	11553 (74.33)	1975 (12.71)	950 (6.11)	511 (3.29)	554 (3.56)
30-34	27108	19000 (70.09)	4365 (16.10)	2219 (8.19)	887 (3.27)	637 (2.35)
35+	22322	14772 (66.18)	4264 (19.10)	2321 (10.40)	632 (2.83)	333 (1.49)
Paternal height (cm)						
<178	16269	11730 (72.10)	2429 (14.93)	1209 (7.43)	478 (2.94)	423 (2.60)
178-180	14756	10442 (70.76)	2315 (15.69)	1235 (8.37)	436 (2.95)	328 (2.22)
181-185	18224	12715 (69.77)	2946 (16.17)	1518 (8.33)	591 (3.24)	454 (2.49)
186+	17763	12136 (68.32)	3030 (17.06)	1561 (8.79)	596 (3.36)	440 (2.48)
Paternal BMI (kg/m2)						
<25	29262	20145 (68.84)	4972 (16.99)	2558 (8.74)	906 (3.10)	681 (2.33)
25-29	30208	21255 (70.36)	4748 (15.72)	2431 (8.05)	991 (3.28)	783 (2.59)
30-35	5819	4322 (74.27)	780 (13.40)	428 (7.36)	157 (2.70)	132 (2.27)
35+	976	764 (78.28)	105 (10.76)	51 (5.23)	27 (2.77)	29 (2.97)
Paternal gross income						
<150k	4227	3082 (72.91)	550 (13.01)	312 (7.38)	140 (3.31)	143 (3.38)
150-199K	2579	1887 (73.17)	365 (14.15)	177 (6.86)	78 (3.02)	72 (2.79)
200-299k	13446	9809 (72.95)	1874 (13.94)	992 (7.38)	400 (2.97)	371 (2.76)
300-399k	21802	15505 (71.12)	3279 (15.04)	1779 (8.16)	687 (3.15)	552 (2.53)
400k+	23157	15429 (66.63)	4391 (18.96)	2134 (9.22)	741 (3.20)	462 (2.00)
Paternal education						
High school (not completed)	6745	5043 (74.77)	795 (11.79)	485 (7.19)	188 (2.79)	234 (3.47)
High school (completed)	25489	18718 (73.44)	3332 (13.07)	1845 (7.24)	803 (3.15)	791 (3.10)
College level	18593	12670 (68.14)	3231 (17.38)	1655 (8.90)	634 (3.41)	403 (2.17)
Postgraduate level	16444	10740 (65.31)	3421 (20.80)	1570 (9.55)	485 (2.95)	228 (1.39)
Paternal smoking in pregnancy (cigs/day)						
Non smoker	51795	36610 (70.68)	8382 (16.18)	4173 (8.06)	1523 (2.94)	1107 (2.14)
<5	7327	4738 (64.66)	1328 (18.12)	715 (9.76)	307 (4.19)	239 (3.26)
5-10	1122	779 (69.43)	167 (14.88)	97 (8.65)	42 (3.74)	37 (3.30)
10+	6956	5022 (72.20)	887 (12.75)	557 (8.01)	223 (3.21)	267 (3.84)
Paternal drugs use in pregnancy						
No	54621	39192 (71.75)	8463 (15.49)	4268 (7.81)	1516 (2.78)	1182 (2.16)
Yes	360	197 (54.72)	71 (19.72)	54 (15.00)	20 (5.56)	18 (5.00)

Supplementary Information, Table S10. Association between confounders and paternal alcohol in 1st trimester pregnancy (in units/occasion) – numbers (%)

	Total	0	<1	1-2	3-4	5+
Year of birth						
1999-2003	12593	598 (4.75)	153 (1.21)	3617 (28.72)	3497 (27.77)	4728 (37.54)
2004-2005	20687	918 (4.44)	237 (1.15)	6356 (30.72)	5569 (26.92)	7607 (36.77)
2006-2007	23196	1146 (4.94)	2659 (11.46)	7132 (30.75)	5513 (23.77)	6746 (29.08)
2008-2009	11622	568 (4.89)	1593 (13.71)	3650 (31.41)	2701 (23.24)	3110 (26.76)
Fetal presentation						
Normal cephalic	61872	2959 (4.78)	4246 (6.86)	18959 (30.64)	15661 (25.31)	20047 (32.40)
Breech	2503	94 (3.76)	184 (7.35)	706 (28.21)	658 (26.29)	861 (34.40)
Transverse	152	8 (5.26)	12 (7.89)	50 (32.89)	42 (27.63)	40 (26.32)
Anomaly cephalic	3026	146 (4.82)	175 (5.78)	854 (28.22)	789 (26.07)	1062 (35.10)
Birth weight (g)						
<2500	1334	64 (4.80)	92 (6.90)	359 (26.91)	310 (23.24)	509 (38.16)
2500-2999	5882	296 (5.03)	407 (6.92)	1733 (29.46)	1429 (24.29)	2017 (34.29)
3000-3499	20035	954 (4.76)	1369 (6.83)	6074 (30.32)	5087 (25.39)	6551 (32.70)
3500-3999	25758	1188 (4.61)	1780 (6.91)	7836 (30.42)	6563 (25.48)	8391 (32.58)
4000+	15089	728 (4.82)	994 (6.59)	4753 (31.50)	3891 (25.79)	4723 (31.30)
Folic acid use around conception						
No	16126	908 (5.63)	852 (5.28)	4548 (28.20)	4004 (24.83)	5814 (36.05)
Yes	49852	2189 (4.39)	3666 (7.35)	15591 (31.27)	12738 (25.55)	15668 (31.43)
Planned pregnancy						
Yes	11621	588 (5.06)	827 (7.12)	3160 (27.19)	2627 (22.61)	4419 (38.03)
No	55734	2578 (4.63)	3772 (6.77)	17382 (31.19)	14467 (25.96)	17535 (31.46)
Parity						
0	32191	1437 (4.46)	1870 (5.81)	8145 (25.30)	7969 (24.76)	12770 (39.67)
1+	34998	1732 (4.95)	2712 (7.75)	12379 (35.37)	9081 (25.95)	9094 (25.98)
Ethnic background						
Norwegian	56229	2348 (4.18)	3645 (6.48)	16824 (29.92)	14367 (25.55)	19045 (33.87)
Other	10599	824 (7.77)	921 (8.69)	3564 (33.63)	2588 (24.42)	2702 (25.49)
Financial strain						
Yes	39280	1947 (4.96)	2655 (6.76)	12300 (31.31)	10143 (25.82)	12235 (31.15)
No	27597	1180 (4.28)	1907 (6.91)	8093 (29.33)	6856 (24.84)	9561 (34.65)
Maternal age (years)						
<25	8285	592 (7.15)	556 (6.71)	1545 (18.65)	1524 (18.39)	4068 (49.10)
25-29	24270	1193 (4.92)	1517 (6.25)	6491 (26.74)	5843 (24.07)	9226 (38.01)
30-34	25626	978 (3.82)	1739 (6.79)	8858 (34.57)	7161 (27.94)	6890 (26.89)
35+	9917	467 (4.71)	830 (8.37)	3861 (38.93)	2752 (27.75)	2007 (20.24)
Maternal height (cm)						
<164	14874	864 (5.81)	1075 (7.23)	4502 (30.27)	3599 (24.20)	4834 (32.50)
164-167	14526	677 (4.66)	1064 (7.32)	4412 (30.37)	3607 (24.83)	4766 (32.81)
168-171	18317	840 (4.59)	1178 (6.43)	5509 (30.08)	4751 (25.94)	6039 (32.97)
172+	19662	798 (4.06)	1288 (6.55)	6123 (31.14)	5136 (26.12)	6317 (32.13)
Maternal pre-preg BMI (kg/m2)						
<20	8426	443 (5.26)	612 (7.26)	2579 (30.61)	2073 (24.60)	2719 (32.27)
20-24	37171	1652 (4.44)	2367 (6.37)	11480 (30.88)	9596 (25.82)	12076 (32.49)
25-29	14437	654 (4.53)	1019 (7.06)	4329 (29.99)	3670 (25.42)	4765 (33.01)
30-35	4648	262 (5.64)	375 (8.07)	1371 (29.50)	1141 (24.55)	1499 (32.25)
35+	1669	100 (5.99)	167 (10.01)	478 (28.64)	364 (21.81)	560 (33.55)
Maternal gross income						
<150k	11218	939 (8.37)	801 (7.14)	3007 (26.81)	2317 (20.65)	4154 (37.03)
150-199K	6782	425 (6.27)	468 (6.90)	1911 (28.18)	1559 (22.99)	2419 (35.67)
200-299k	22183	984 (4.44)	1342 (6.05)	6672 (30.08)	5616 (25.32)	7569 (34.12)
300-399k	17525	504 (2.88)	1318 (7.52)	5660 (32.30)	4837 (27.60)	5206 (29.71)
400k+	8144	190 (2.33)	556 (6.83)	2823 (34.66)	2433 (29.87)	2142 (26.30)
Maternal education						
High school (not completed)	4770	369 (7.74)	321 (6.73)	1122 (23.52)	990 (20.75)	1968 (41.26)
High school (completed)	17744	865 (4.87)	1227 (6.92)	4522 (25.48)	4045 (22.80)	7085 (39.93)
College level	27941	1333 (4.77)	1888 (6.76)	8700 (31.14)	7219 (25.84)	8801 (31.50)
Postgraduate level	16226	573 (3.53)	1117 (6.88)	6015 (37.07)	4666 (28.76)	3855 (23.76)
Maternal smoking in pregnancy (cigs/day)						
Non smoker	64265	3107 (4.83)	4427 (6.89)	19916 (30.99)	16396 (25.51)	20419 (31.77)
<2	1711	50 (2.92)	105 (6.14)	363 (21.22)	380 (22.21)	813 (47.52)

2-4	452	10 (2.21)	14 (3.10)	98 (21.68)	106 (23.45)	224 (49.56)
4+	1266	45 (3.55)	72 (5.69)	266 (21.01)	289 (22.83)	594 (46.92)
Maternal drugs use in pregnancy						
No	56991	2805 (4.92)	3987 (7.00)	17894 (31.40)	14483 (25.41)	17822 (31.27)
Yes	109	5 (4.59)	8 (7.34)	18 (16.51)	18 (16.51)	60 (55.05)
Paternal age (years)						
<25	3103	223 (7.19)	203 (6.54)	520 (16.76)	488 (15.73)	1669 (53.79)
25-29	15543	863 (5.55)	967 (6.22)	3661 (23.55)	3409 (21.93)	6643 (42.74)
30-34	27108	1100 (4.06)	1685 (6.22)	8383 (30.92)	7204 (26.58)	8736 (32.23)
35+	22322	1043 (4.67)	1782 (7.98)	8186 (36.67)	6173 (27.65)	5138 (23.02)
Paternal height (cm)						
<178	16269	943 (5.80)	1196 (7.35)	4929 (30.30)	4088 (25.13)	5113 (31.43)
178-180	14756	696 (4.72)	1010 (6.84)	4556 (30.88)	3695 (25.04)	4799 (32.52)
181-185	18224	742 (4.07)	1287 (7.06)	5585 (30.65)	4594 (25.21)	6016 (33.01)
186+	17763	782 (4.40)	1128 (6.35)	5359 (30.17)	4621 (26.01)	5873 (33.06)
Paternal BMI (kg/m2)						
<25	29262	1518 (5.19)	1973 (6.74)	9677 (33.07)	7386 (25.24)	8708 (29.76)
25-29	30208	1251 (4.14)	2035 (6.74)	8750 (28.97)	7904 (26.17)	10268 (33.99)
30-35	5819	294 (5.05)	480 (8.25)	1522 (26.16)	1315 (22.60)	2208 (37.94)
35+	976	54 (5.53)	109 (11.17)	246 (25.20)	202 (20.70)	365 (37.40)
Paternal gross income						
<150k	4227	464 (10.98)	327 (7.74)	1056 (24.98)	813 (19.23)	1567 (37.07)
150-199K	2579	233 (9.03)	204 (7.91)	674 (26.13)	540 (20.94)	928 (35.98)
200-299k	13446	810 (6.02)	810 (6.02)	3765 (28.00)	3092 (23.00)	4969 (36.96)
300-399k	21802	897 (4.11)	1616 (7.41)	6704 (30.75)	5485 (25.16)	7100 (32.57)
400k+	23157	597 (2.58)	1594 (6.88)	7734 (33.40)	6664 (28.78)	6568 (28.36)
Paternal education						
High school (not completed)	6745	443 (6.57)	509 (7.55)	1631 (24.18)	1423 (21.10)	2739 (40.61)
High school (completed)	25489	1228 (4.82)	1728 (6.78)	6669 (26.16)	5922 (23.23)	9942 (39.01)
College level	18593	898 (4.83)	1206 (6.49)	6008 (32.31)	4980 (26.78)	5501 (29.59)
Postgraduate level	16444	598 (3.64)	1155 (7.02)	6225 (37.86)	4725 (28.73)	3741 (22.75)
Paternal smoking in pregnancy (cigs/day)						
Non smoker	51795	2781 (5.37)	3842 (7.42)	17182 (33.17)	13378 (25.83)	14612 (28.21)
<5	7327	142 (1.94)	305 (4.16)	1697 (23.16)	1900 (25.93)	3283 (44.81)
5-10	1122	38 (3.39)	76 (6.77)	237 (21.12)	261 (23.26)	510 (45.45)
10+	6956	214 (3.08)	370 (5.32)	1428 (20.53)	1529 (21.98)	3415 (49.09)
Paternal drugs use in pregnancy						
No	54621	2922 (5.35)	3974 (7.28)	17552 (32.13)	13939 (25.52)	16234 (29.72)
Yes	360	3 (0.83)	11 (3.06)	46 (12.78)	70 (19.44)	230 (63.89)

Supplementary Information, Figure S1 Distribution of confounders by parental alcohol use BEFORE the pregnancy - cumulative distribution functions. X-axis represents median of category

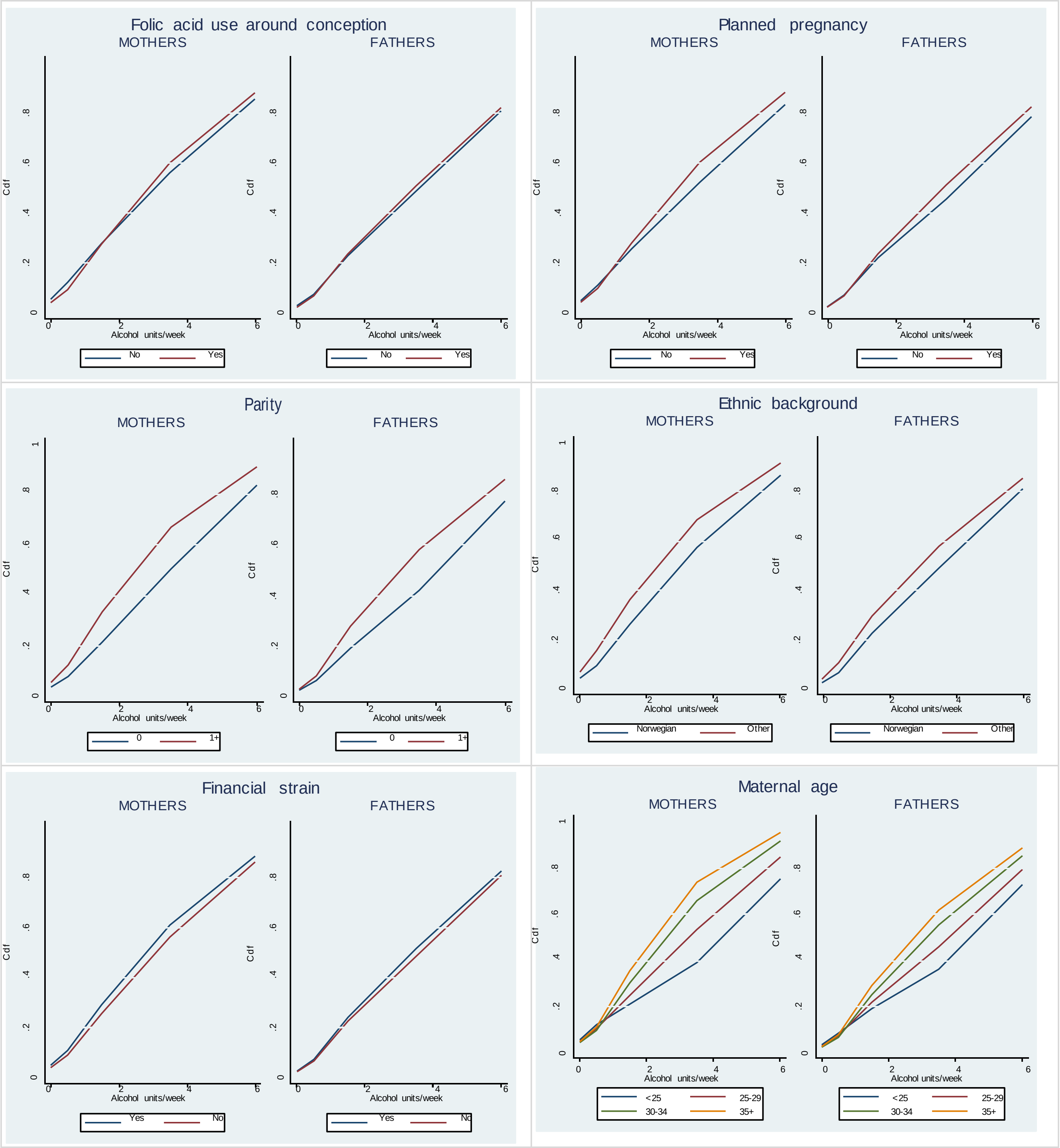

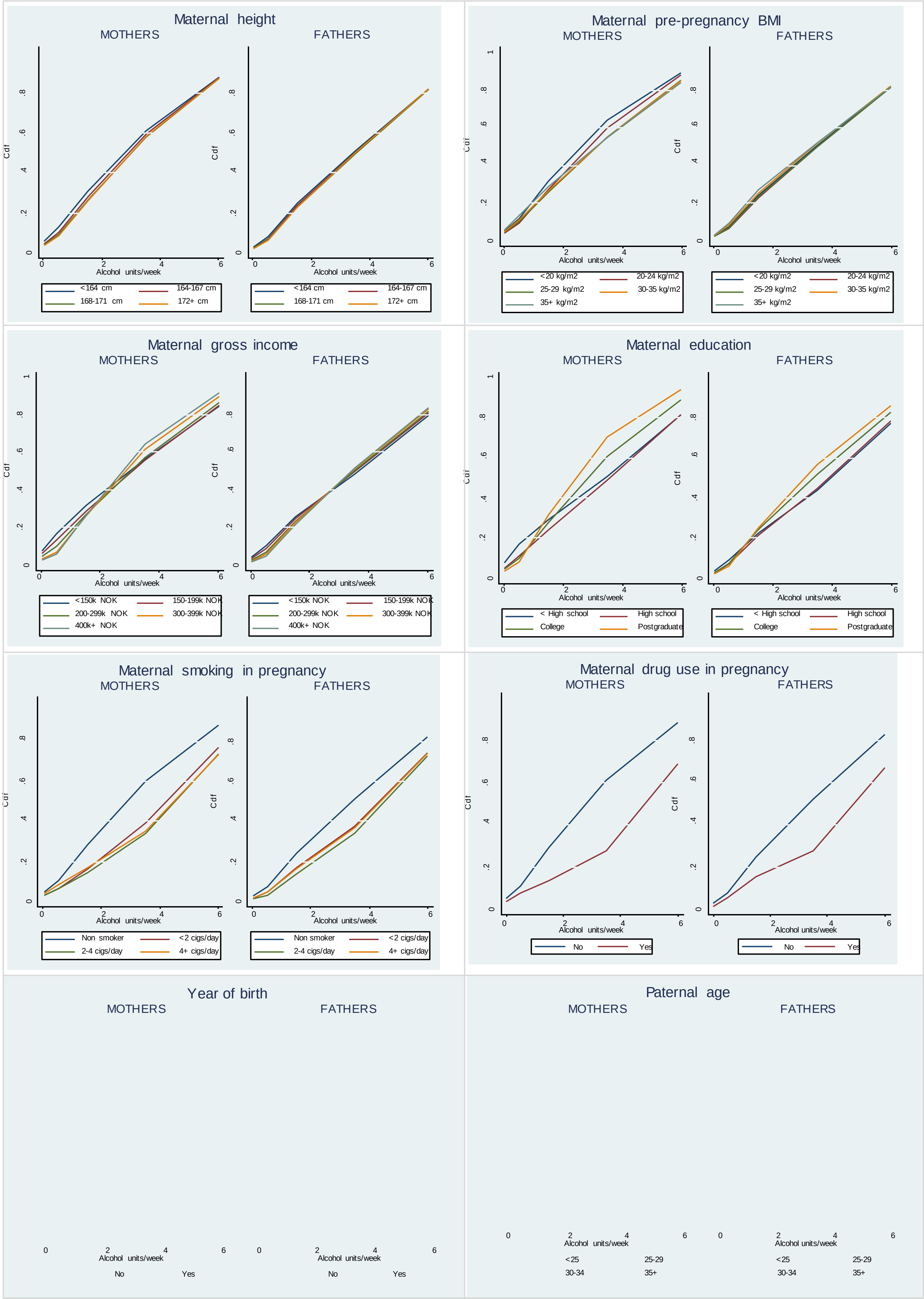
Maternal height
MOTHERS
FATHERS
Cdf
Alcohol units/week
<164 cm
164-167 cm
168-171 cm
172+ cm
Maternal pre-pregnancy BMI
MOTHERS
FATHERS
<20 kg/m2
20-24 kg/m2
25-29 kg/m2
30-35 kg/m2
35+ kg/m2
Maternal gross income
MOTHERS
FATHERS
<150k NOK
150-199k NOK
200-299k NOK
300-399k NOK
400k+ NOK
Maternal education
MOTHERS
FATHERS
< High school
High school
College
Postgraduate
Maternal smoking in pregnancy
MOTHERS
FATHERS
Non smoker
<2 cigs/day
2-4 cigs/day
4+ cigs/day
Maternal drug use in pregnancy
MOTHERS
FATHERS
No
Yes
Year of birth
MOTHERS
FATHERS
Paternal age
MOTHERS
FATHERS
<25
25-29
30-34
35+

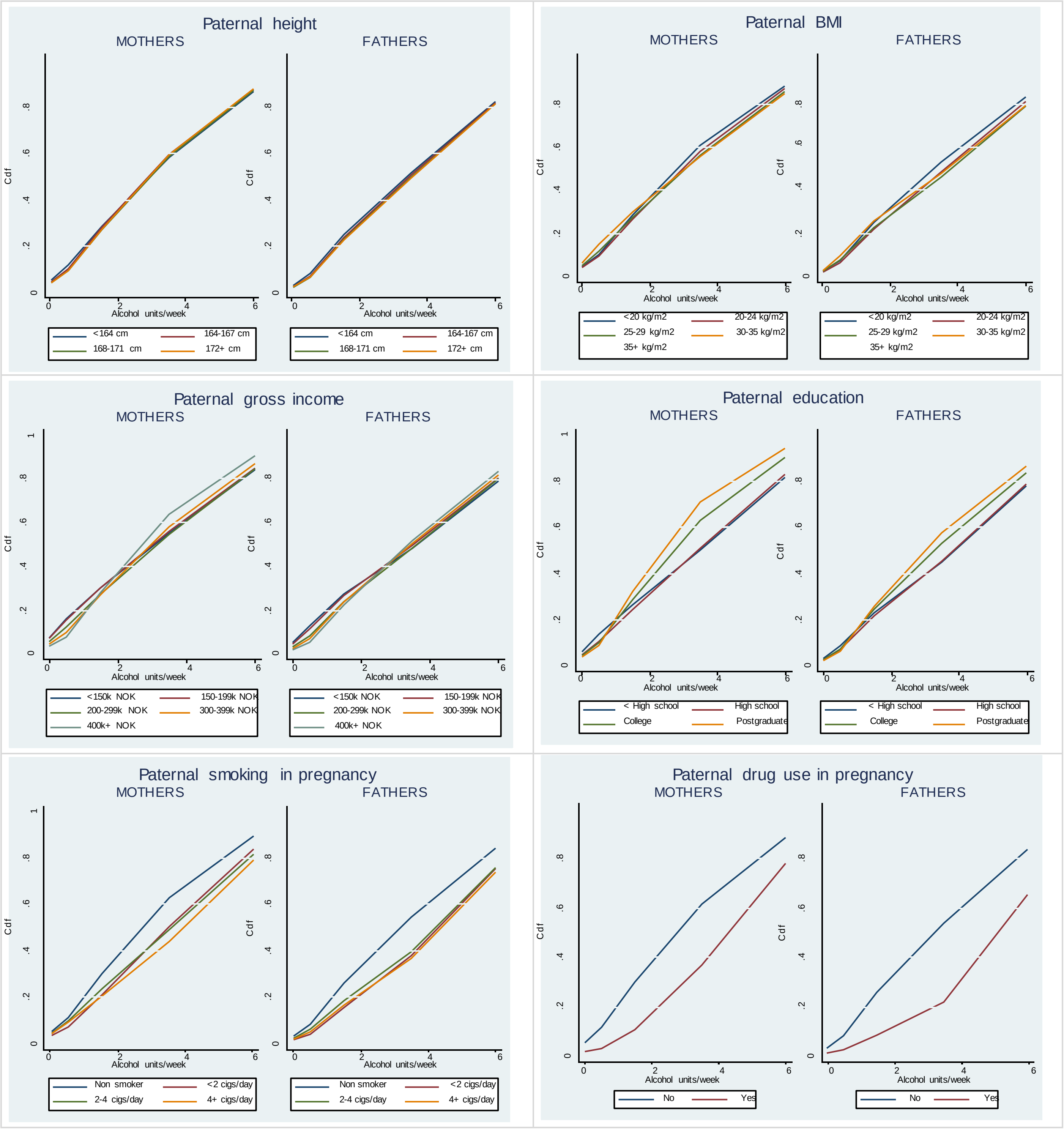
Paternal height
MOTHERS
FATHERS
Cdf
Alcohol units/week
<164 cm
164-167 cm
168-171 cm
172+ cm
Paternal BMI
MOTHERS
FATHERS
<20 kg/m2
20-24 kg/m2
25-29 kg/m2
30-35 kg/m2
35+ kg/m2
Paternal gross income
MOTHERS
FATHERS
<150k NOK
150-199k NOK
200-299k NOK
300-399k NOK
400k+ NOK
Paternal education
MOTHERS
FATHERS
< High school
High school
College
Postgraduate
Paternal smoking in pregnancy
MOTHERS
FATHERS
Non smoker
<2 cigs/day
2-4 cigs/day
4+ cigs/day
Paternal drug use in pregnancy
MOTHERS
FATHERS
No
Yes

Supplementary Information, Figure S2 Distribution of confounders by parental alcohol use DURING the pregnancy - cumulative distribution functions. X-axis represents median of category

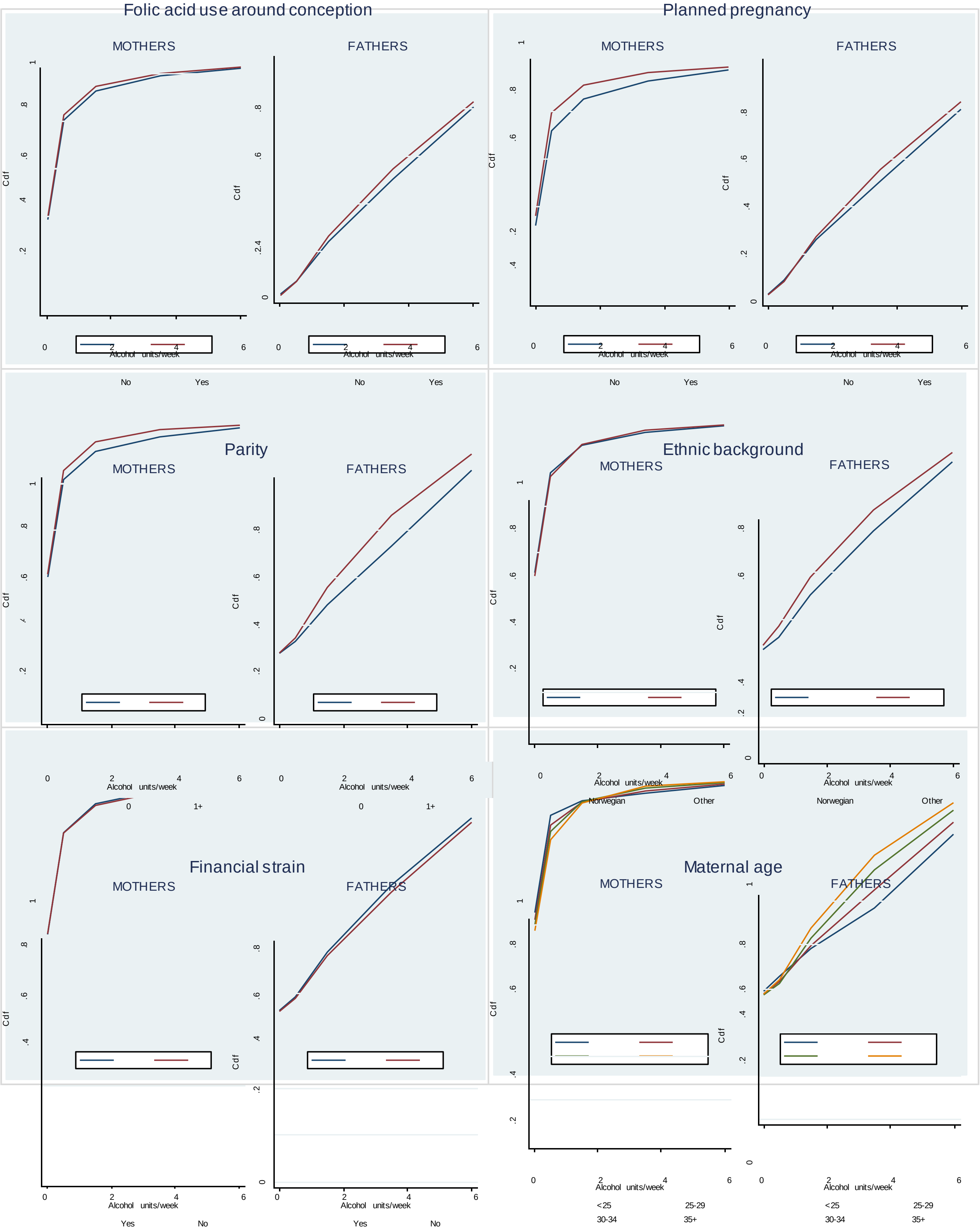

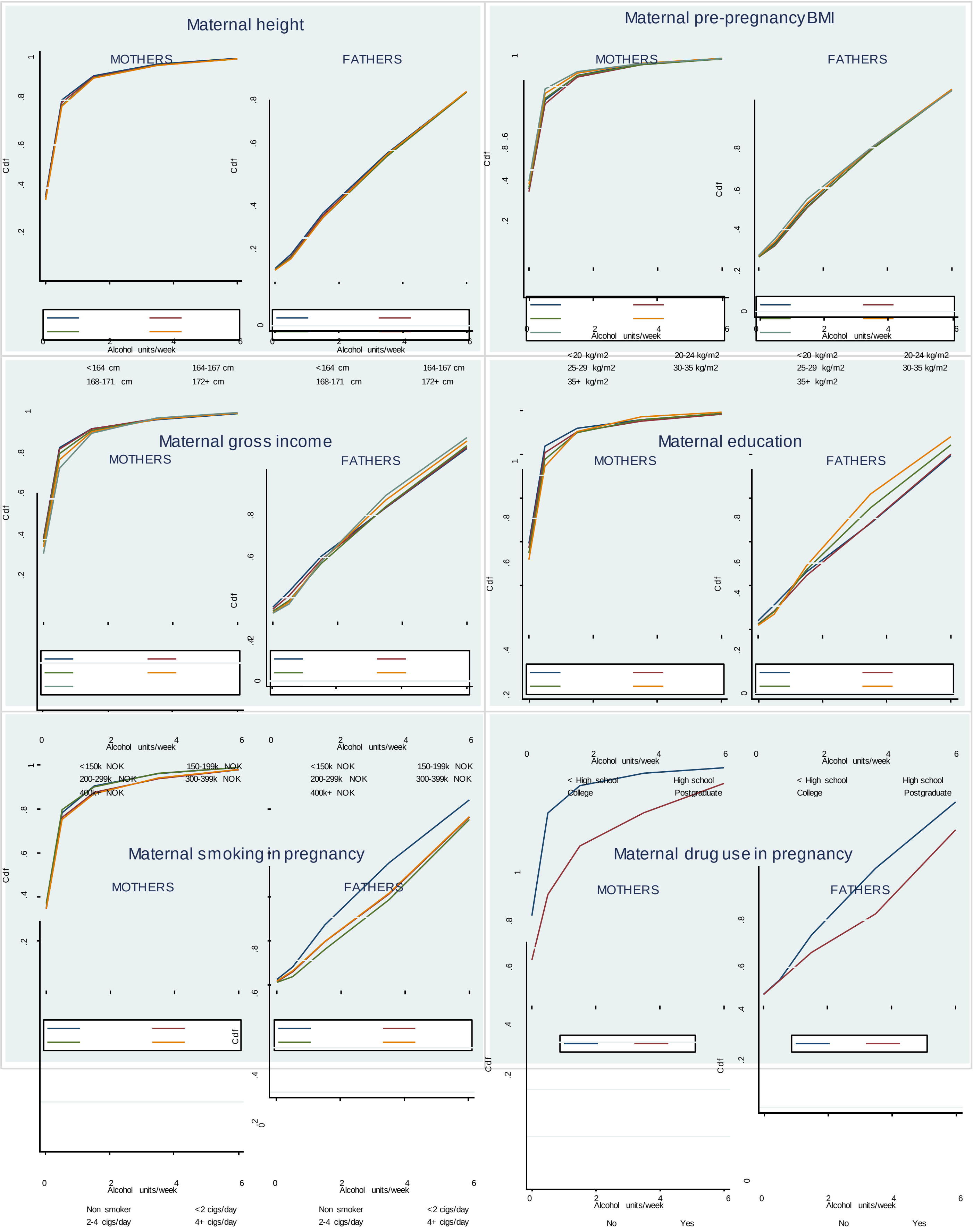
Maternal height
MOTHERS
FATHERS
Cdf
Alcohol units/week
<164 cm
164-167 cm
168-171 cm
172+ cm
Maternal pre-pregnancy BMI
<20 kg/m2
20-24 kg/m2
25-29 kg/m2
30-35 kg/m2
35+ kg/m2
Maternal gross income
<150k NOK
150-199k NOK
200-299k NOK
300-399k NOK
400k+ NOK
Maternal education
< High school
High school
College
Postgraduate
Maternal smoking in pregnancy
Non smoker
<2 cigs/day
2-4 cigs/day
4+ cigs/day
Maternal drug use in pregnancy
No
Yes

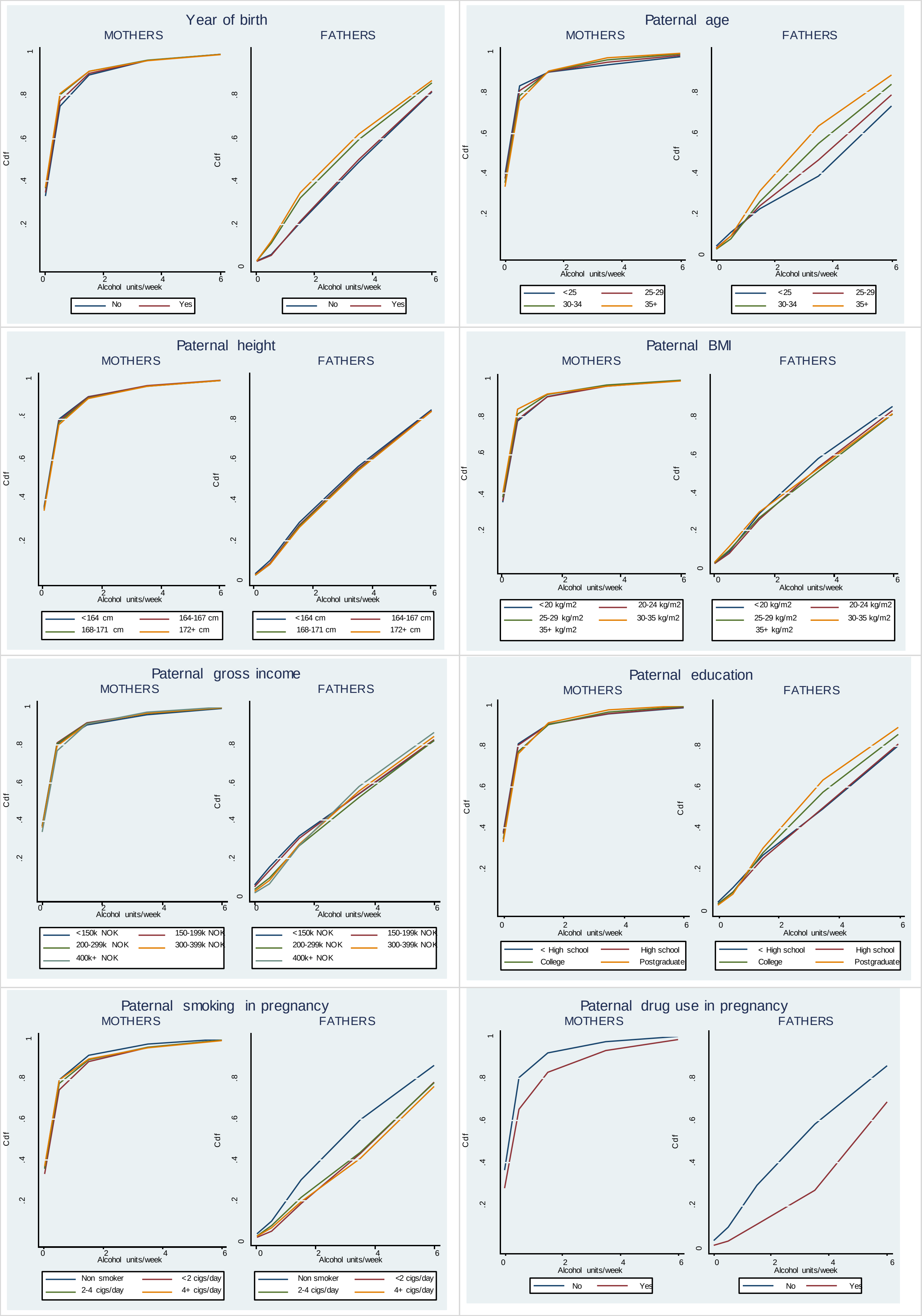
Year of birth
MOTHERS
FATHERS
Cdf
Alcohol units/week
No
Yes
Paternal age
<25
25-29
30-34
35+
Paternal height
<164 cm
164-167 cm
168-171 cm
172+ cm
Paternal BMI
<20 kg/m2
20-24 kg/m2
25-29 kg/m2
30-35 kg/m2
35+ kg/m2
Paternal gross income
<150k NOK
150-199k NOK
200-299k NOK
300-399k NOK
400k+ NOK
Paternal education
< High school
High school
College
Postgraduate
Paternal smoking in pregnancy
Non smoker
<2 cigs/day
2-4 cigs/day
4+ cigs/day
Paternal drug use in pregnancy

Confounders-outcomes associations

Supplementary Information, Table S11. Association between confounders and head circumference at birth - betas are expressed in units of sex-standardised head circumference (differences in standard deviations compared to the reference category).

	beta	95% CI	pvalue
Birth weight (100g)	0.11	0.11, 0.11	<0.001
Year of birth (2years)	-0.03	-0.04, -0.02	<0.001
Maternal age (5years)	0.04	0.03, 0.05	<0.001
Maternal height (5cm)	0.09	0.09, 0.10	<0.001
Maternal pre-preg weight (5kg)	0.06	0.06, 0.06	<0.001
Maternal smoking (2cigs/day)	-0.04	-0.05, -0.04	<0.001
Paternal age (5years)	0.03	0.02, 0.03	<0.001
Paternal height (5cm)	0.05	0.05, 0.06	<0.001
Paternal pre-preg weight (5kg)	0.03	0.03, 0.03	<0.001
Paternal smoking (2cigs/day)	-0.01	-0.01, -0.00	<0.001
Fetal presentation (ref Normal cephalic)			
Breech	0.18	0.15, 0.21	<0.001
Transverse	0.04	-0.10, 0.17	0.588
Anomaly cephalic	-0.07	-0.10, -0.03	<0.001
Folic acid use	-0.01	-0.03, 0.00	0.073
Planned pregnancy (yes Vs no)	0.03	0.01, 0.05	0.001
Parity (1+ vs no previous pregnancies)	0.21	0.20, 0.22	<0.001
Financial strain (yes vs no)	0.01	-0.01, 0.02	0.385
Ethnic background (other vs Norwegian)	-0.14	-0.15, -0.12	<0.001
Maternal gross income (ref 200-299k)	beta	CI	pvalue
<150k	-0.02	-0.04, -0.01	0.011
150-199K	0.02	0.00, 0.05	0.022
300-399k	-0.01	-0.03, 0.00	0.136
400k+	-0.02	-0.04, -0.00	0.029
Maternal education (ref College level)			
High school (not completed	-0.05	-0.08, -0.03	<0.001
High school (completed)	0.01	-0.00, 0.03	0.090
Postgraduate level	-0.01	-0.03, 0.00	0.052
Maternal smoking in preg (yes vs no)	-0.12	-0.14, -0.09	<0.001
Maternal drugs use (yes vs no)	-0.25	-0.41, -0.09	0.002
Paternal gross income (ref 200-299k)			
<150k	-0.06	-0.09, -0.03	<0.001
150-199K	-0.03	-0.07, -0.00	0.049
300-399k	0.03	0.01, 0.04	<0.001
400k+	-0.01	-0.03, -0.00	0.034
Paternal education (ref College level)			
High school (not completed	-0.01	-0.03, 0.01	0.359
High school (completed)	0.01	-0.00, 0.02	0.080
Postgraduate level	-0.02	-0.04, -0.01	0.007
Paternal smoking in preg (yes vs no)	-0.03	-0.04, -0.01	0.001
Paternal drugs use (yes vs no)	-0.16	-0.24, -0.07	<0.001

Supplementary Information, Table S12. Association between confounders and head circumference at 3 months - betas are expressed in units of head circumference standardised for sex and age at clinic visit (differences in standard deviations compared to the reference category)..

	beta	95% CI	pvalue
Birth weight (100g)	0.09	0.09, 0.09	<0.001
Year of birth (2years)	-0.01	-0.02, -0.00	0.044
Maternal age (5years)	0.03	0.02, 0.04	<0.001
Maternal height (5cm)	0.10	0.09, 0.11	<0.001
Maternal pre-preg weight (5kg)	0.05	0.04, 0.05	<0.001
Maternal smoking (2cigs/day)	-0.04	-0.05, -0.03	<0.001
Paternal age (5years)	0.02	0.01, 0.03	<0.001
Paternal height (5cm)	0.08	0.07, 0.08	<0.001
Paternal pre-preg weight (5kg)	0.04	0.03, 0.04	<0.001
Paternal smoking (2cigs/day)	-0.01	-0.01, -0.01	<0.001
Fetal presentation (ref Normal cephalic)			
Breech	-0.04	-0.08, 0.01	0.085
Transverse	-0.08	-0.25, 0.09	0.355
Anomaly cephalic	0.11	0.07, 0.15	<0.001
Folic acid use	0.02	-0.00, 0.04	0.119
Planned pregnancy (yes Vs no)	0.03	0.00, 0.05	0.026
Parity (1+ vs no previous pregnancies)	0.12	0.10, 0.14	<0.001
Financial strain (yes vs no)	-0.00	-0.02, 0.01	0.799
Ethnic background (other vs Norwegian)	-0.10	-0.13, -0.08	<0.001
Maternal gross income (ref 200-299k)			
<150k	-0.02	-0.05, -0.00	0.040
150-199K	-0.02	-0.05, 0.01	0.187
300-399k	0.02	-0.00, 0.04	0.069
400k+	0.05	0.02, 0.07	<0.001
Maternal education (ref College level)			
High school (not completed	-0.11	-0.14, -0.07	<0.001
High school (completed)	-0.05	-0.07, -0.03	<0.001
Postgraduate level	0.06	0.04, 0.08	<0.001
Maternal smoking in preg (yes vs no)	-0.10	-0.13, -0.07	<0.001
Maternal drugs use (yes vs no)	-0.31	-0.52, -0.10	0.004
Paternal gross income (ref 200-299k)			
<150k	-0.01	-0.05, 0.02	0.432
150-199K	-0.03	-0.07, 0.01	0.192
300-399k	0.01	-0.01, 0.03	0.170
400k+	0.01	-0.01, 0.02	0.469
Paternal education (ref College level)			
High school (not completed	-0.06	-0.09, -0.03	<0.001
High school (completed)	-0.04	-0.05, -0.02	<0.001
Postgraduate level	0.06	0.04, 0.08	<0.001
Paternal smoking in preg (yes vs no)	-0.05	-0.07, -0.03	<0.001
Paternal drugs use (yes vs no)	-0.10	-0.21, 0.02	0.099

Sensitivity analyses

Supplementary Information, Table S13. Maternal and Paternal Average Alcohol Units per Week and Head Circumference at Birth – Differences in SD Scores (Beta) and 95% Confidence Intervals (CI), MoBa data, Norway, 1999-2009.

Before pregnancy

		Mother				Father	
	Beta	95% CI	*P*		Beta	95% CI	*P*
Crude model							
Non drinker	Ref				Ref		
<1 unit	0.02	-0.01, 0.05	0.248		0.01	-0.03, 0.05	0.632
1-2 units	-0.01	-0.04, 0.02	0.453		0.02	-0.02, 0.06	0.350
3-4 units	-0.02	-0.05, 0.02	0.352		-0.02	-0.06, 0.03	0.456
5+ units	-0.06	-0.09, -0.02	0.002		-0.05	-0.10, -0.01	0.011
Mutually adjusted model							
Non drinker	Ref				Ref		
<1 unit	0.02	-0.02, 0.05	0.356		0.01	-0.04, 0.05	0.738
1-2 units	-0.01	-0.04, 0.03	0.672		0.02	-0.02, 0.07	0.327
3-4 units	-0.00	-0.04, 0.04	0.969		-0.01	-0.06, 0.04	0.702
5+ units	-0.03	-0.07, 0.01	0.128		-0.04	-0.09, 0.01	0.108
Fully and mutually adjusted model							
Non drinker	Ref				Ref		
<1 unit	0.00	-0.03, 0.04	0.790		-0.01	-0.06, 0.03	0.599
1-2 units	0.01	-0.03, 0.04	0.755		0.01	-0.04, 0.05	0.684
3-4 units	0.01	-0.03, 0.05	0.542		-0.00	-0.05, 0.04	0.899
5+ units	0.01	-0.03, 0.05	0.495		-0.01	-0.06, 0.03	0.594

Test for trend maternal and paternal alcohol intake in full model: p=0.369 and p=0.425

In the first trimester of gestation

		Mother				Father	
	Beta	95% CI	*P*		Beta	95% CI	*P*
Crude model							
Non drinker	Ref				Ref		
<1 unit	-0.00	-0.02, 0.02	0.863		0.01	-0.02, 0.05	0.468
1-2 units	-0.04	-0.09, 0.02	0.202		0.03	-0.01, 0.06	0.187
3-4 units	-0.11	-0.20, -0.01	0.030		-0.00	-0.04, 0.04	0.846
5+ units	-0.06	-0.18, 0.05	0.299		-0.03	-0.07, 0.01	0.190
Mutually adjusted model							
Non drinker	Ref				Ref		
<1 unit	0.00	-0.02, 0.02	0.783		0.02	-0.02, 0.05	0.424
1-2 units	-0.03	-0.09, 0.02	0.244		0.03	-0.01, 0.07	0.178
3-4 units	-0.10	-0.19, -0.00	0.049		-0.00	-0.04, 0.04	0.976
5+ units	-0.04	-0.16, 0.07	0.468		-0.02	-0.06, 0.02	0.280
Fully and mutually adjusted model							
Non drinker	Ref				Ref		
<1 unit	0.00	-0.02, 0.02	0.847		-0.00	-0.04, 0.03	0.881
1-2 units	-0.00	-0.06, 0.05	0.906		0.02	-0.02, 0.06	0.367
3-4 units	-0.05	-0.14, 0.05	0.337		0.01	-0.03, 0.05	0.768
5+ units	0.02	-0.09, 0.13	0.753		0.00	-0.04, 0.04	0.976

Test for trend maternal and paternal alcohol intake in full model: p=0.977 and p=0.879

Supplementary Information, Table S14. Maternal and Paternal Average Alcohol Units per Week and Head Circumference at 3 Months Post-Partum – Differences in SD Scores (Beta) and 95% Confidence Intervals (CI), MoBa data, Norway, 1999-2009.

Before pregnancy

		Mother				Father	
	Beta	95% CI	*P*		Beta	95% CI	*P*
Crude model							
Non drinker	Ref				Ref		
<1 unit	-0.00	-0.04, 0.04	0.947		-0.04	-0.09, 0.01	0.144
1-2 units	-0.00	-0.04, 0.04	0.831		-0.02	-0.08, 0.03	0.393
3-4 units	0.02	-0.02, 0.06	0.386		-0.04	-0.10, 0.01	0.140
5+ units	-0.01	-0.05, 0.04	0.820		-0.04	-0.10, 0.01	0.121
Mutually adjusted model							
Non drinker	Ref				Ref		
<1 unit	0.02	-0.02, 0.06	0.361		-0.06	-0.12, 0.00	0.063
1-2 units	0.02	-0.03, 0.07	0.402		-0.04	-0.10, 0.02	0.182
3-4 units	0.05	-0.00, 0.10	0.072		-0.07	-0.13, -0.00	0.043
5+ units	0.03	-0.03, 0.08	0.359		-0.07	-0.13, -0.00	0.037
Fully and mutually adjusted model							
Non drinker	Ref				Ref	Ref	
<1 unit	0.01	-0.04, 0.05	0.719		-0.05	-0.11, 0.01	0.082
1-2 units	0.02	-0.03, 0.06	0.491		-0.04	-0.10, 0.02	0.245
3-4 units	0.03	-0.02, 0.08	0.243		-0.05	-0.11, 0.02	0.149
5+ units	0.04	-0.02, 0.09	0.181		-0.04	-0.11, 0.02	0.187

Test for trend maternal and paternal alcohol intake in full model: p=0.110 and p=0.903

In the first trimester of gestation

		Mother				Father	
	Beta	95% CI	*P*		Beta	95% CI	*P*
Crude model							
Non drinker	Ref				Ref		
<1 unit	0.02	-0.00, 0.04	0.114		-0.05	-0.10, 0.00	0.074
1-2 units	-0.02	-0.09, 0.05	0.632		-0.02	-0.07, 0.03	0.392
3-4 units	0.03	-0.09, 0.16	0.583		-0.02	-0.08, 0.03	0.369
5+ units	-0.13	-0.28, 0.02	0.088		-0.05	-0.11, 0.00	0.054
Mutually adjusted model							
Non drinker	Ref				Ref		
<1 unit	0.02	-0.00, 0.05	0.077		-0.05	-0.10, 0.00	0.061
1-2 units	-0.02	-0.09, 0.05	0.627		-0.02	-0.08, 0.03	0.358
3-4 units	0.03	-0.09, 0.16	0.581		-0.03	-0.08, 0.03	0.313
5+ units	-0.12	-0.27, 0.03	0.124		-0.06	-0.11, -0.00	0.045
Fully and mutually adjusted model							
Non drinker	Ref				Ref		
<1 unit	0.02	-0.01, 0.04	0.183		-0.05	-0.10, -0.00	0.044
1-2 units	0.00	-0.07, 0.07	0.927		-0.03	-0.08, 0.03	0.324
3-4 units	0.06	-0.06, 0.18	0.320		-0.02	-0.07, 0.03	0.496
5+ units	-0.08	-0.23, 0.07	0.280		-0.04	-0.10, 0.01	0.105

Test for trend maternal and paternal alcohol intake in full model: p=0.842 and p=0.995

Supplementary Information, Table S15. Maternal and Paternal Average Alcohol Units per Week and Microcephaly at Birth – Odds Ratio (OR) and 95% Confidence Intervals (CI), MoBa data, Norway, 1999-2009.

Before pregnancy

		Mother				Father	
	OR	95% CI	*P*		OR	95% CI	*P*
Crude model							
Non drinker	Ref				Ref		
<1 unit	0.74	0.51, 1.05	0.093		1.21	0.66, 2.20	0.536
1-2 units	0.80	0.56, 1.16	0.242		1.14	0.63, 2.10	0.661
3-4 units	1.06	0.71, 1.56	0.783		1.46	0.80, 2.68	0.220
5+ units	1.07	0.71, 1.62	0.736		1.81	1.00, 3.29	0.051
Mutually adjusted model							
Non drinker	Ref				Ref		
<1 unit	0.62	0.42, 0.91	0.015		1.56	0.82, 2.96	0.173
1-2 units	0.63	0.42, 0.95	0.029		1.49	0.76, 2.88	0.243
3-4 units	0.75	0.48, 1.17	0.209		1.90	0.97, 3.70	0.061
5+ units	0.71	0.45, 1.14	0.161		2.28	1.17, 4.44	0.016
Fully and mutually adjusted model							
Non drinker	Ref				Ref		
<1 unit	0.64	0.43, 0.94	0.025		1.66	0.87, 3.15	0.123
1-2 units	0.61	0.40, 0.93	0.020		1.55	0.80, 3.02	0.196
3-4 units	0.73	0.47, 1.15	0.175		1.90	0.97, 3.72	0.061
5+ units	0.63	0.39, 1.02	0.058		2.25	1.15, 4.41	0.018

Test for trend maternal and paternal alcohol intake in full model: p=0.795 and p=0.010

In the first trimester of gestation

		Mother				Father	
	OR	95% CI	*P*		OR	95% CI	*P*
Crude model							
Non drinker	Ref				Ref		
<1 unit	0.87	0.69, 1.11	0.264		1.07	0.65, 1.78	0.785
1-2 units	1.15	0.61, 2.17	0.658		1.05	0.63, 1.76	0.852
3-4 units	2.24	0.99, 5.07	0.053		1.15	0.68, 1.96	0.596
5+ units	1.07	0.27, 4.35	0.920		1.59	0.94, 2.68	0.083
Mutually adjusted model							
Non drinker	Ref				Ref		
<1 unit	0.83	0.65, 1.05	0.126		1.10	0.66, 1.82	0.726
1-2 units	1.05	0.56, 1.99	0.871		1.08	0.64, 1.82	0.769
3-4 units	1.98	0.87, 4.49	0.104		1.20	0.71, 2.05	0.497
5+ units	0.92	0.23, 3.74	0.908		1.61	0.95, 2.73	0.079
Fully and mutually adjusted model							
Non drinker	Ref				Ref		
<1 unit	0.83	0.65, 1.06	0.140		1.18	0.71, 1.98	0.523
1-2 units	0.99	0.52, 1.88	0.967		1.14	0.67, 1.93	0.625
3-4 units	1.81	0.79, 4.14	0.163		1.22	0.71, 2.10	0.467
5+ units	0.79	0.19, 3.23	0.741		1.63	0.95, 2.80	0.077

Test for trend maternal and paternal alcohol intake in full model: p=0.981 and p=0.013

Supplementary Information, Table S16. Maternal and Paternal Average Alcohol Units per Week and Microcephaly at 3 Months Post-Partum – Odds Ratio (OR) and 95% Confidence Intervals (CI), MoBa data, Norway, 1999-2009.

Before pregnancy

		Mother				Father	
	OR	95% CI	*P*		OR	95% CI	*P*
Crude model							
Non drinker	Ref				Ref		
<1 unit	1.10	0.83, 1.44	0.520		1.35	0.88, 2.08	0.175
1-2 units	0.98	0.73, 1.30	0.867		1.34	0.87, 2.07	0.185
3-4 units	1.06	0.77, 1.45	0.714		1.49	0.96, 2.32	0.072
5+ units	1.13	0.81, 1.56	0.466		1.30	0.84, 2.02	0.239
Mutually adjusted model							
Non drinker	Ref				Ref		
<1 unit	0.95	0.71, 1.29	0.750		1.42	0.89, 2.25	0.140
1-2 units	0.83	0.60, 1.14	0.244		1.41	0.88, 2.27	0.157
3-4 units	0.90	0.64, 1.28	0.561		1.60	0.98, 2.59	0.058
5+ units	1.00	0.69, 1.44	0.982		1.36	0.83, 2.23	0.217
Fully and mutually adjusted model							
Non drinker	Ref				Ref		
<1 unit	1.00	0.74, 1.35	0.996		1.42	0.89, 2.27	0.137
1-2 units	0.85	0.61, 1.17	0.312		1.41	0.87, 2.27	0.162
3-4 units	0.95	0.66, 1.35	0.763		1.56	0.96, 2.53	0.074
5+ units	0.97	0.67, 1.41	0.875		1.33	0.81, 2.18	0.255

Test for trend maternal and paternal alcohol intake in full model: p=0.804 and p=0.604

In the first trimester of gestation

		Mother				Father	
	OR	95% CI	*P*		OR	95% CI	*P*
Crude model							
Non drinker	Ref				Ref		
<1 unit	0.85	0.71, 1.01	0.061		1.22	0.84, 1.78	0.290
1-2 units	0.89	0.53, 1.50	0.669		1.15	0.79, 1.69	0.462
3-4 units	0.96	0.39, 2.32	0.921		1.23	0.83, 1.82	0.291
5+ units	2.27	1.12, 4.63	0.024		1.21	0.81, 1.80	0.356
Mutually adjusted model							
Non drinker	Ref				Ref		
<1 unit	0.84	0.70, 1.00	0.047		1.24	0.86, 1.81	0.253
1-2 units	0.89	0.53, 1.49	0.657		1.19	0.81, 1.75	0.373
3-4 units	0.95	0.39, 2.31	0.905		1.27	0.86, 1.89	0.229
5+ units	2.24	1.09, 4.58	0.027		1.25	0.84, 1.88	0.269
Fully and mutually adjusted model							
Non drinker	Ref				Ref		
<1 unit	0.83	0.70, 1.00	0.046		1.30	0.89, 1.90	0.176
1-2 units	0.81	0.48, 1.37	0.438		1.23	0.84, 1.82	0.291
3-4 units	0.87	0.36, 2.14	0.768		1.30	0.87, 1.94	0.203
5+ units	1.93	0.94, 3.99	0.075		1.29	0.86, 1.95	0.222

Test for trend maternal and paternal alcohol intake in full model: p=0.343 and p=0.910

Supplementary Information, Table S17. Associations of paternal alcohol drinking before pregnancy and microcephaly– odds ratio (OR) of microcephaly and 95% confidence intervals (CI) by categories of average alcohol dose per occasion, with several exclusion criteria.

a) excluding all congenital malformations at birth

	At birth			At 3 months		
	OR	95% CI	p	OR	95% CI	p
Fully and mutually adjusted model						
Non drinker	Ref			Ref		
<1 drink	1.80	0.87, 3.72	0.113	1.56	0.90, 2.69	0.112
1-2 drinks	1.41	0.73, 2.70	0.308	1.35	0.83, 2.20	0.229
3-4 drinks	1.62	0.83, 3.12	0.155	1.60	0.98, 2.62	0.062
5+ drinks	1.83	0.95, 3.52	0.070	1.56	0.95, 2.54	0.079

b) excluding Maternal pre-eclampsia, gestational diabetes, or unknown follow-up

	At birth			At 3 months		
	OR	95% CI	p	OR	95% CI	p
Fully and mutually adjusted model						
Non drinker						
<1 drink	1.62	0.78, 3.40	0.199	1.59	0.93, 2.71	0.090
1-2 drinks	1.37	0.71, 2.65	0.348	1.25	0.77, 2.02	0.369
3-4 drinks	1.43	0.71, 0.65	0.292	1.47	0.91, 2.39	0.118
5+ drinks	1.70	0.74, 2.78	0.114	1.53	0.95, 2.49	0.083

c) excluding outliers HC more than 4 sd away, or mother-reported 'abnormal HC'

	At birth			At 3 months		
	OR	95% CI	p	OR	95% CI	p
Fully and mutually adjusted model						
Non drinker						
<1 drink	1.76	0.85, 3.65	0.130	1.49	0.88, 2.53	0.141
1-2 drinks	1.48	0.77, 2.83	0.242	1.20	0.75, 1.93	0.451
3-4 drinks	1.64	0.85, 3.16	0.141	1.38	0.86, 2.23	0.182
5+ drinks	1.96	1.02, 3.76	0.043	1.44	0.90, 2.32	0.131

d) excluding C-sections

	At birth			At 3 months		
	OR	95% CI	p	OR	95% CI	p
Fully and mutually adjusted model						
Non drinker						
<1 drink	2.45	0.99, 6.08	0.053	1.44	0.82, 2.52	0.204
1-2 drinks	1.79	0.77, 4.14	0.173	1.12	0.68, 1.85	0.659
3-4 drinks	2.02	0.87, 4.70	0.102	1.32	0.79, 2.19	0.284
5+ drinks	2.32	1.00, 5.35	0.049	1.44	0.87, 2.38	0.157

e) excluding mothers at risk or problem drinking (relative to year prior to pregnancy)

	At birth			At 3 months		
	OR	95% CI	p	OR	95% CI	p
Fully and mutually adjusted model						
Non drinker	Ref			Ref		
<1 drink	1.82	0.85, 3.89	0.125	1.60	0.94, 2.75	0.085
1-2 drinks	1.65	0.84, 3.25	0.149	1.34	0.83, 2.16	0.239
3-4 drinks	1.78	0.90, 3.54	0.098	1.61	0.99, 2.61	0.055
5+ drinks	2.07	1.05, 4.08	0.037	1.49	0.91, 2.42	0.110

Supplementary Information, Table S18. Associations of paternal alcohol drinking before pregnancy and microcephaly– odds ratio (OR) of microcephaly and 95% confidence intervals (CI) by categories of average alcohol dose per occasion, including all 68,098 eligible trios.

	At birth			At 3 months		
	OR	95% CI	p	OR	95% CI	p
Crude model						
Non drinker						
<1 drink	1.20	0.71, 2.03	0.499	1.33	0.88, 2.00	0.172
1-2 drinks	0.86	0.55, 1.34	0.516	1.16	0.82, 1.63	0.402
3-4 drinks	1.01	0.65, 1.57	0.956	1.34	0.95, 1.89	0.095
5+ drinks	1.33	0.87, 2.04	0.189	1.37	0.98, 1.91	0.070
Mutually adjusted model						
Non drinker						
<1 drink	1.34	0.78, 2.33	0.292	1.41	0.92, 2.15	0.113
1-2 drinks	1.00	0.62, 1.63	0.996	1.24	0.86, 1.81	0.255
3-4 drinks	1.13	0.69, 1.84	0.627	1.44	0.99, 2.09	0.059
5+ drinks	1.44	0.89, 2.33	0.138	1.51	1.04, 2.19	0.031

Supplementary Information, Table S19. Associations of paternal alcohol drinking before pregnancy and microcephaly– odds ratio (OR) of microcephaly and 95% confidence intervals (CI) by categories of average alcohol dose per occasion, with additional adjustment for fetal presentation at birth.

	At birth			At 3 months		
	OR	95% CI	p	OR	95% CI	p
Fully and mutually adjusted model						
Non drinker						
<1 drink	1.80	0.87, 3.71	0.114	1.61	0.95, 2.71	0.075
1-2 drinks	1.46	0.76, 2.81	0.254	1.25	0.78, 2.00	0.357
3-4 drinks	1.62	0.84, 3.13	0.148	1.45	0.90, 2.33	0.125
5+ drinks	1.90	0.99, 3.65	0.053	1.49	0.93, 2.39	0.100
